# Extracellular vesicles released from macrophages modulates interleukin-1β in astrocytic and neuronal cells

**DOI:** 10.1038/s41598-023-29746-y

**Published:** 2023-02-21

**Authors:** Sunitha Kodidela, Namita Sinha, Asit Kumar, Lina Zhou, Sandip Godse, Santosh Kumar

**Affiliations:** Department of Pharmaceutical Sciences, College of Pharmacy, The University of Tennessee Health Science Center, Memphis, TN 38163 USA

**Keywords:** Biochemistry, Immunology, Microbiology, Neuroscience, Diseases, Pathogenesis

## Abstract

We have recently demonstrated that long-term exposure of cigarette smoke condensate (CSC) to HIV-uninfected (U937) and -infected (U1) macrophages induce packaging of pro-inflammatory molecules, particularly IL-1β, in extracellular vesicles (EVs). Therefore, we hypothesize that exposure of EVs derived from CSC-treated macrophages to CNS cells can increase their IL-1β levels contributing to neuroinflammation. To test this hypothesis, we treated the U937 and U1 differentiated macrophages once daily with CSC (10 µg/ml) for 7 days. Then, we isolated EVs from these macrophages and treated these EVs with human astrocytic (SVGA) and neuronal (SH-SY5Y) cells in the absence and presence of CSC. We then examined the protein expression of IL-1β and oxidative stress related proteins, cytochrome P450 2A6 (CYP2A6), superoxide dismutase-1 (SOD1), catalase (CAT). We observed that the U937 cells have lower expression of IL-1β compared to their respective EVs, confirming that most of the produced IL-1β are packaged into EVs. Further, EVs isolated from HIV-infected and uninfected cells, both in the absence and presence of CSC, were treated to SVGA and SH-SY5Y cells. These treatments showed a significant increase in the levels of IL-1β in both SVGA and SH-SY5Y cells. However, under the same conditions, the levels of CYP2A6, SOD1, and catalase were only markedly altered. These findings suggest that the macrophages communicate with astrocytes and neuronal cells via EVs-containing IL-1β in both HIV and non-HIV setting and could contribute to neuroinflammation.

## Introduction

Macrophages are important innate immune cells that act as the initial line of defense against invading pathogens. Macrophages are activated by various stimuli and signals resulting in the release of cytokines leading to exacerbation or amelioration of inflammation^1,2^. Typically, pro- and anti-inflammatory actions of macrophages are balanced in a precise manner^2,3^. On the other hand, abnormal macrophage responses can trigger immunological abnormalities and uncontrolled inflammation linked to a variety of diseases^3–6^. For instance, HIV infection to macrophages is known to cause inflammation and oxidative stress, which exacerbates pathogenesis^7–10^. Further, macrophages serve as viral reservoirs during HIV infection and spread the virus to brain perivascular macrophages and microglia leading to HIV-associated neurocognitive disorders (HAND)^9,11–13^. Activated macrophages and/or microglia release viral proteins, cytokines/chemokines, and/or oxidative stress factors that are taken up by astrocytes and/or neuronal cells, and subsequently cause inflammation in these brain cells^9,14,15^.

Extracellular vesicles (EVs) have recently emerged as important vehicles/carriers for cell–cell communication^10,16–22^. They can package and transport diverse biological cargos such as proteins, mRNA, miRNA, and small molecules to local or distance cells^23–29^. EVs can also serve as communicators between peripheral and brain cells via plasma circulation and by crossing the blood–brain barrier (BBB)^30–32^. Macrophage and/or microglial EVs, especially upon HIV infection, may package and release inflammatory and/or oxidative factors into plasma and/or cerebrospinal fluid (CSF), resulting in transfer to astrocytes and/or neurons. The role of macrophage EVs in various disease states has been widely investigated, and increasing evidence suggests that these EVs play key roles in disease progression^33,34^. However, the role of EVs derived from macrophages in cell–cell interactions in the brain, leading to neuroinflammation has not been examined.

The prevalence of tobacco smoking is increased in HIV-infected populations ^35,36^, and is known to exacerbate HIV pathogenesis and HAND^37–39^. Previously we have shown that tobacco constituents, cigarette smoke condensate (CSC), nicotine, and/or benzo(a)pyrene (BaP) increase HIV pathogenesis via cytochrome P450-mediated oxidative stress pathway^38–40^. More recently, we have demonstrated that monocyte-derived EVs, upon exposure to CSC, can influence HIV replication in macrophages by cell–cell interaction^41^. We have also shown that EVs derived from HIV-infected and uninfected macrophages differentially package cytokines and antioxidant enzymes (AOEs) upon exposure to CSC^20^, subsequently affecting HIV pathogenesis.

In the current study, we examined whether EVs derived from HIV-infected and uninfected macrophages, especially upon exposure to CSC, contribute to inflammation in the brain cells. We hypothesize that EVs derived from HIV-infected and uninfected macrophages, especially upon exposure to CSC, will transfer inflammatory cytokines, particularly interleukin-1β (IL-1β), to astrocytes and neuronal cells, thus contributing to inflammation in the brain.

## Results

### Characterizations of EVs from HIV-uninfected U937 and HIV-infected U1 macrophages

**Figure 1:**
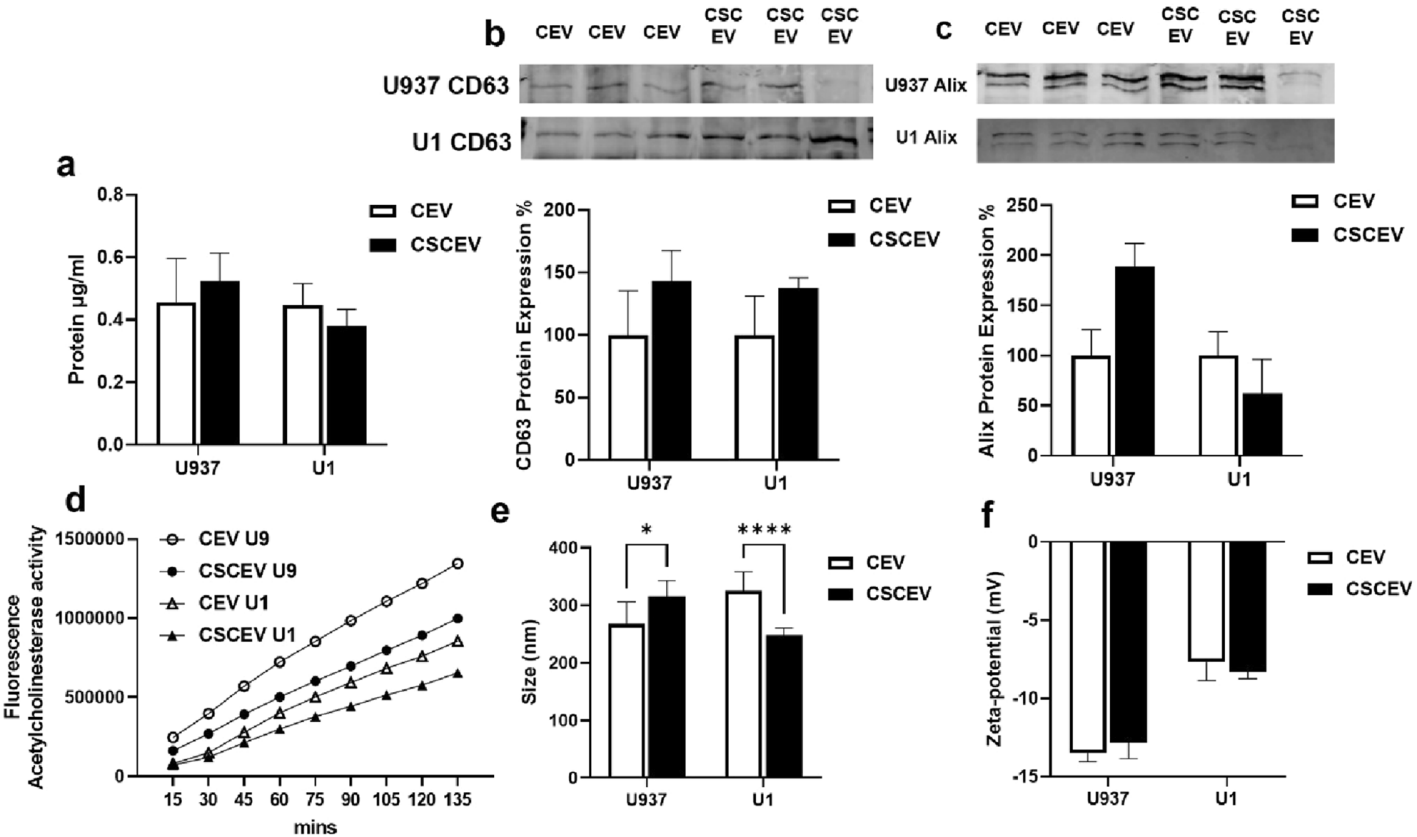
Characterizations of EVs isolated from HIV-uninfected U937 and HIV-infected U1 macrophages after 7 days of treatment with CSC. (**a**) Protein quantification of EVs. (**b**, **c**) Western blot for EV marker proteins CD63 and Alix using control for each cell as 100%. (**d**) Acetylcholinesterase (AChE) activity in EVs. The activity was measured at different time intervals using arbitrary fluorescence unit. (**e**) Determination of particle size using DLS (Dynamic Light Scattering). EVs derived from U937: Ctrl: 267.2 ± 38.6 nm, CSC treated 316 ± 26.8 nm; EVs derived from U1: Ctrl: 325.4 ± 33.1 nm, CSC treated 247.5 ± 13.4 nm (Mean ± SD). (**f**) Determination of Zeta-potential of EVs. EVs derived from U937: Ctrl: − 13.5 ± 0.5 mV, CSC treated − 12.9 ± 1.0 mV; EVs derived from U1: Ctrl: − 7.7 ± 1.2 mV, CSC treated − 8.3 ± 0.4 mV (Mean ± SD). All the measurements were done in triplicates. CEV represents EVs isolated from the control group; CSCEV represents EVs isolated from the CSC-treatment group. One-way ANOVA was used to find the significance between the control and CSC groups; * indicates *p*-value < 0.05; *** indicates *p*-value < 0.001.

We have previously published the characterizations of EVs derived from HIV-uninfected U937 and HIV-infected U1 macrophages using Zetasizer, qNano gold, acetylcholine esterase activity, Western blot, and transmission election microspcopy^20,27,41,47^. The EVs isolated from HIV-infected U1 macrophages does not contain detectable virus. In this study, we further characterized EVs with respect to protein amount, EV marker proteins and enzymes activity, and EV size and zeta potential (Fig. 1) as described in the previous studies^20,27,41,47^. We also analyzed whether CSC treatment causes any effect on EV characteristics in U937 and U1 macrophages. As shown in Fig. 1a, there is no significant difference in protein amount between CSC treatment and control groups in both the cells. The level of EV marker CD63 also did not significantly alter upon CSC treatment in both U937 and U1 macrophages (Fig. 1b). However, the level of Alix in U937, but not in U1 macrophages, was significantly increased upon CSC treatment (Fig. 1c). We also measured the acetylcholine esterase activity, another EV marker. The results did not show significant change in the enzyme activity upon CSC treatment (Fig. 1d). However, there was a trend with decrease in activity upon CSC treatment in both the cells. The size and zeta potential of the EVs were within the same range as published earlier^27,47^. Moreover, the size of EVs was significantly increased upon CSC treatment in U937 macrophages, while it was decreased in U1 macrophages (Fig. 1e). However, the zeta potential of EVs upon CSC treatment isolated from both the cells were similar (Fig. 1f). In this study, we did not compare EV characteristics between U937 and U1 macrophages, because the experiments with each cell were done independently. Overall, these results suggest no major changes in the EV characteristics upon CSC treatment in either U937 or U1 macrophages.

### Extracellular vesicles derived from HIV-uninfected U937 Macrophages modulate systemic cytokines in SVG astrocyte cells

Recently, we have shown differential modulation of various cytokines in the absence and presence of CSC in both HIV-uninfected U937 macrophage- and HIV-infected U1 macrophage-derived EVs^20^. Now, we seek to examine whether these EVs modulate cytokines in astrocytes (SVGA cells) in the absence and presence of CSC.

**Figure 2:**
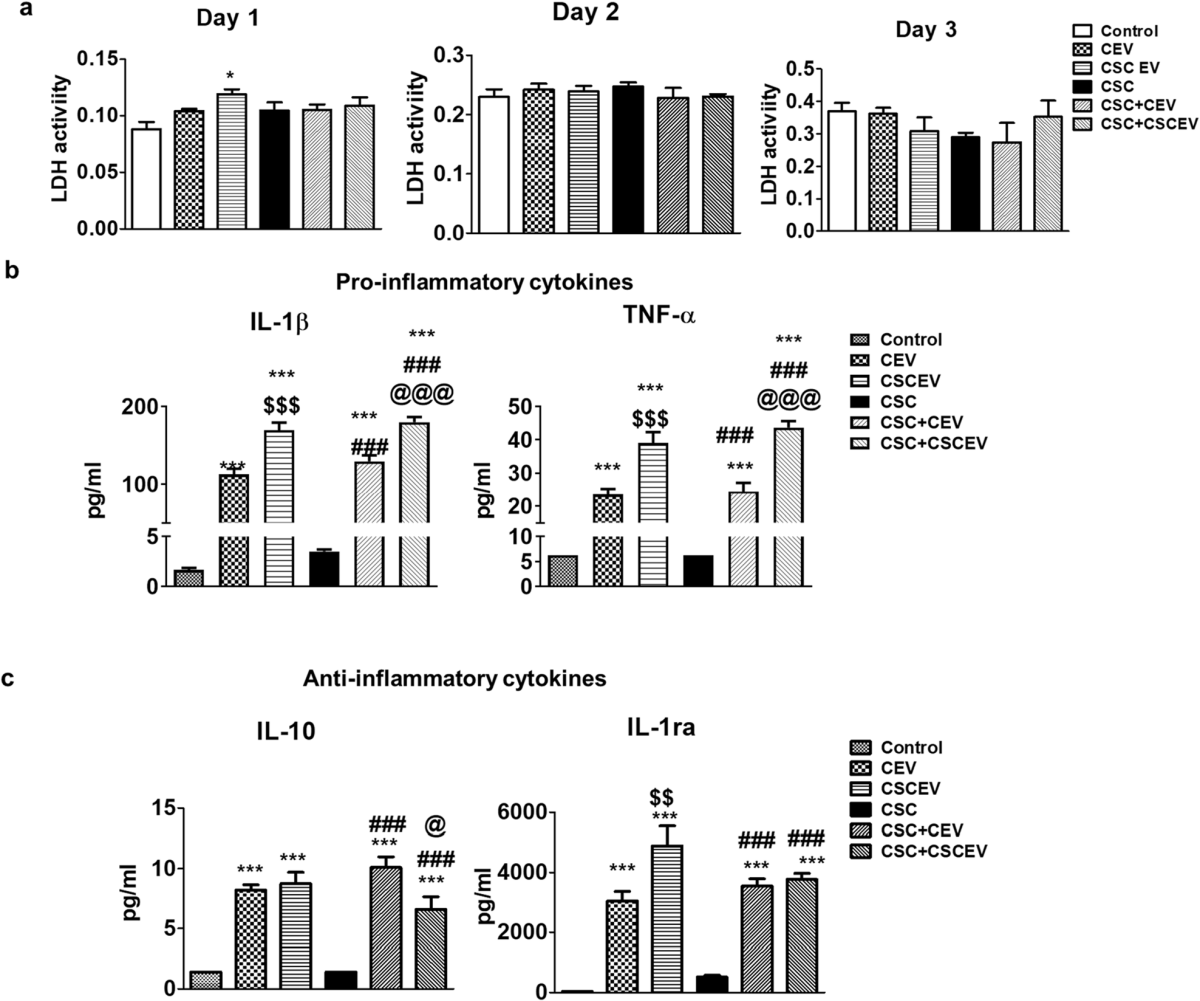
Effect of EVs derived from HIV-uninfected U937 macrophages on cytotoxicity and modulation of cytokines in SVGA cells. The EVs were isolated from U937 macrophages exposed to CSC (10 µg/ml) once daily for 7 days. SVGA cells (0.3 million cells/well/12 well plate) were treated with CSCEV once daily for three days. After three days of treatment, the lactate dehydrogenase (LDH) (**a**) was measured in the supernatant every day, and cytokines were measured on day 3 samples (**b** & **c**). One-way ANOVA with Tukey’s multiple comparison test was used to find the significance among the groups. CEV represents EVs isolated from the control group; CSCEV represents EVs isolated from the CSC-treatment group. *, ** and *** represent *p* < 0.05, *p* ≤ 0.01, and *p* ≤ 0.001 respectively when compared to control; #,##, and ### represents *p* < 0.05, *p* ≤ 0.01, and *p* ≤ 0.001 respectively when compared to CSC; $,$$, and $$$ represents *p* < 0.05, *p* ≤ 0.01, and *p* ≤ 0.001 respectively when compared to CEV; @@@ represents *p* ≤ 0.001 when compared CSC + CEV vs CSC + CSCEV.

Before analyzing the cytokines, we performed an LDH assay to study the cytotoxicity of EVs on the SVGA cells. We observed no significant toxicity in most treatment regimens and time-point, except for CSCEV-treated SVGA on day 1 (Fig. 2a).

Next, we measured cytokine levels in the media of EVs-treated SVGA cells. We observed that both the SVGA cells that received control EV (CEV) and EVs isolated upon CSC treatment (CSCEV) showed elevated levels of IL-β in the media (Fig. 2b). Moreover, the IL-1β levels were higher in the group that received EVs from CSC-exposed macrophages than those from the control group. However, the IL-1β was not elevated further when EVs were given along with CSC exposure to the SVGA cells. This was expected as CSC exposure did not increase the IL-1β level in the SVGA. A similar trend was observed with another proinflammatory cytokine, tumor necrosis factor-alpha (TNF-α).

Furthermore, we also observed that the anti-inflammatory cytokine (Fig. 2c) levels in the SVGA were also high that received EVs from control or CSC-exposed macrophages. However, the IL-10 levels were decreased when the CSCEV were given along with CSC to SVGA cells. On the contrary, the IL-1RA levels were increased when the CEV was given along with CSC to SVGA cells. Overall, the results suggest that the exposure of macrophage-derived EVs increases the levels of proinflammatory cytokines in astrocytic cells, which is further enhanced when the EVs are derived from CSC-exposed macrophages.

### Extracellular vesicles derived from HIV-uninfected U937 macrophages enhance cellular interleukin-1β in SVG astrocyte cells

**Figure 3:**
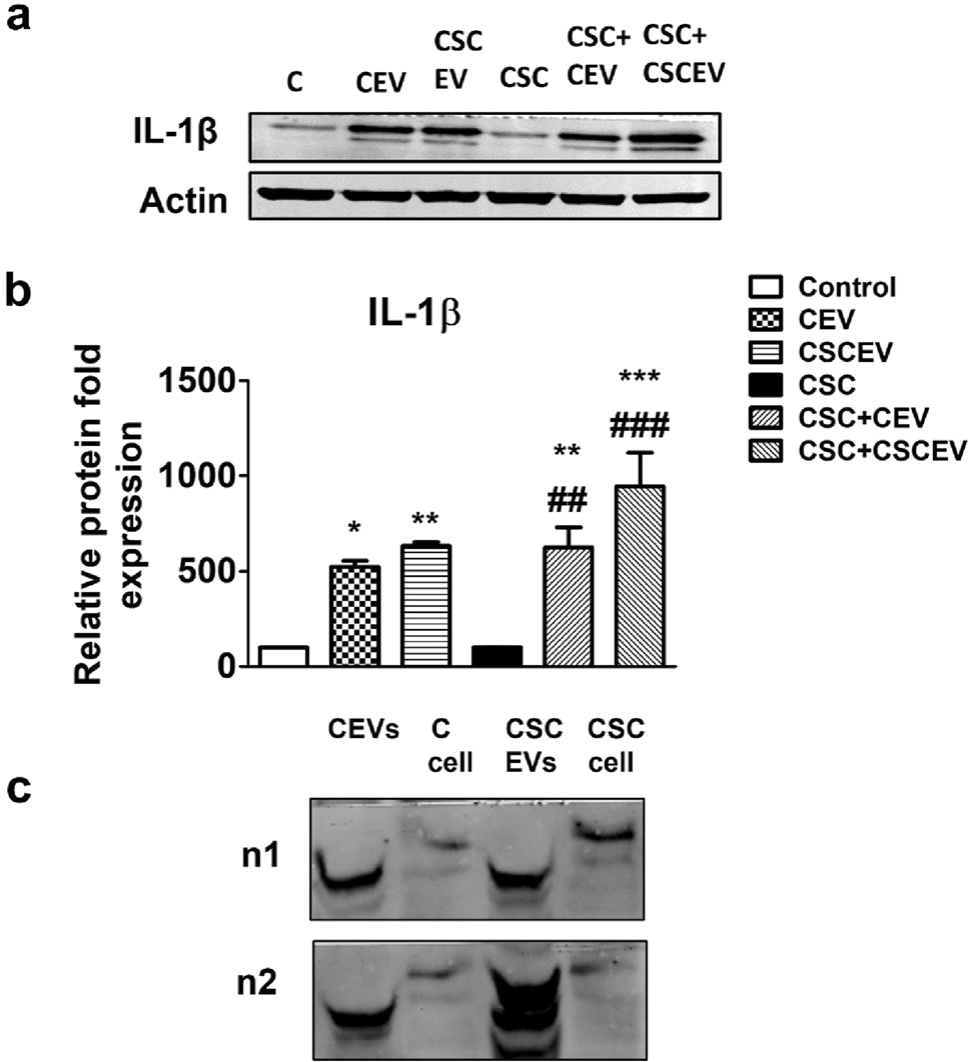
Effect of EVs derived from HIV-uninfected U937 macrophage on IL-1β protein expression in SVGA cells and comparison of IL-1β levels between EVs derived from U937 macrophages and their respective cells. We treated U937 differentiated macrophages (0.8 million cells/well/6wells) with control (DMSO) and CSC (10 µg/ml) once daily for seven days. We harvested the cells and isolated EVs from the media. The SVGA cells (0.1 million cells/well/12well plate) were treated with EVs derived from macrophages once daily for 3 days. After 3 days of treatments, Western blot was performed with an equal amount of protein loaded (5 µg of protein) to measure the expression of IL-1β levels. One-way ANOVA with Tukey’s multiple comparison test was used to find the significance among the groups. **CE** represents EVs isolated from the control group; **CSCEV** represents EVs isolated from the CSC-treatment group; **C-cell** represents control U937 cells; **CSC-cell** represents CSC treated U937 cells. ‘**n**’ represents the number of replicates. *, **, and *** represent *p* < 0.05, *p* ≤ 0.01, and *p* ≤ 0.001 respectively when compared to control; #,##, and ### represents *p* < 0.05, *p* ≤ 0.01, and *p* ≤ 0.001 respectively when compared to CSC.

IL-1β is one of the most important proinflammatory cytokine, and plays a role in the pathology of many diseases^42–46^. Therefore, we examined whether the levels of cellular IL-1β are also modulated by the exposure of CEV and CSCEV in the absence and presence of CSC. Similar to the results in Fig. 2b, the IL-1β protein expression was also higher in the cells that received EVs compared to those that received no EVs in both control and CSC groups. Although not statistically significant, the levels of IL-1β in SVGA cells were further increased when treated with EVs from CSC exposed, compared to control, U937 cells, especially in the presence of CSC. The findings on the cellular modulation of IL-1β (Fig. 3a,b; Supplementary Fig. S2) verify the results on IL-1β obtained from the U937 cell media (Fig. 2b).

In an earlier study, we have shown almost complete packaging of IL-1β in EVs derived from plasma, compared to free IL-1β in plasma, in rats that were self-administered with nicotine^47^. Thus, we wanted to examine whether IL-1β is highly packaged in macrophage-derived EVs and secreted into media. We compared the IL-1β levels between U937 cells and EVs derived from these cells. We found that the expression of IL-1β was higher in EVs than in their respective cells (Fig. 3c; Supplementary Fig. S2), suggesting that most of the IL-1β produced in the cells is packaged in EVs and released in the media. The finding is significant from the context that EVs play significant role in cell–cell communication, macrophages to astrocytes in this case.

### Increased IL-β levels in SVGA cells upon exposure to macrophage-derived EVs are not associated with CYP2A6-mediated oxidative stress pathways

**Figure 4:**
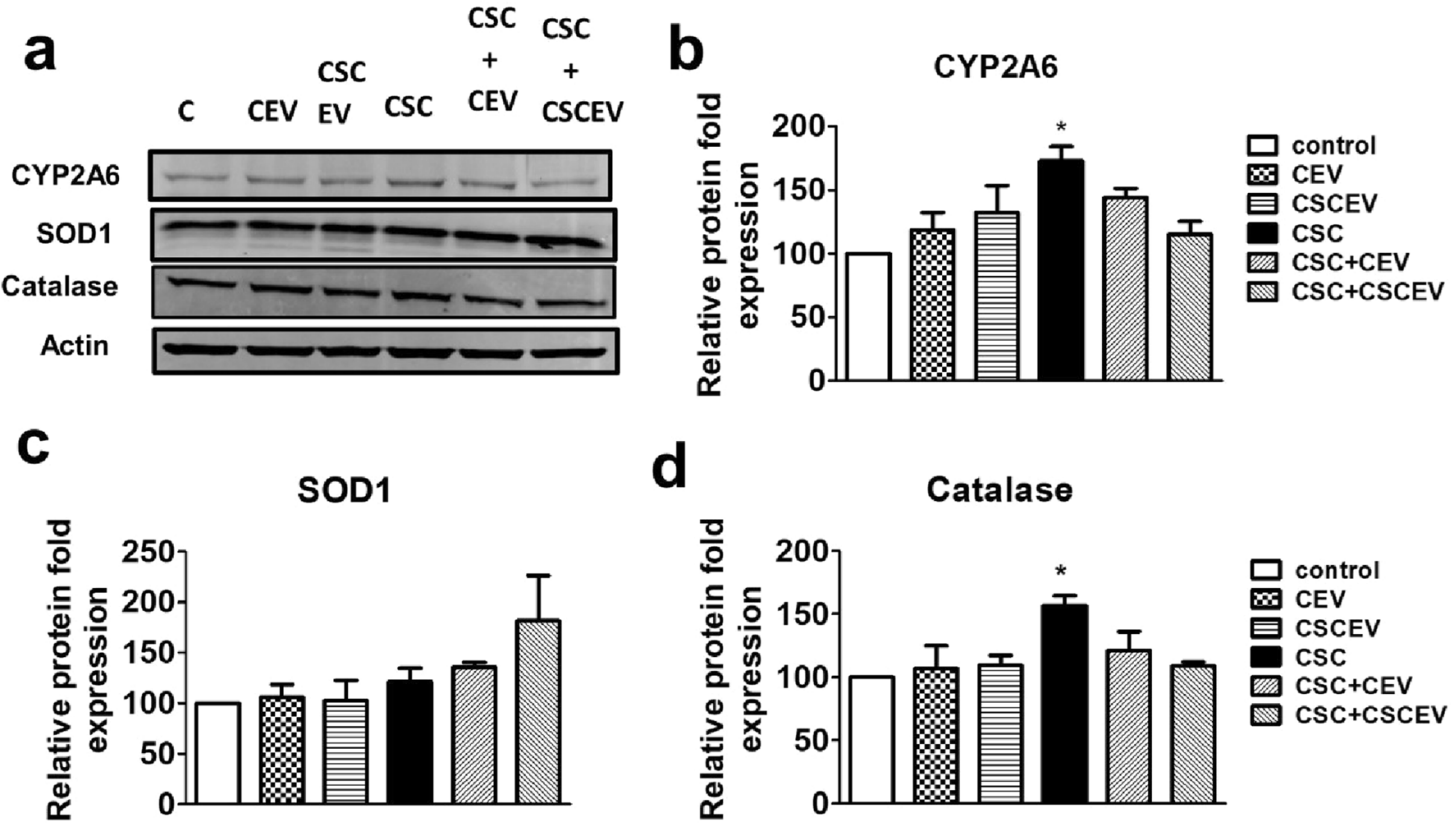
Expression of CYP2A6 and antioxidant enzymes in SVGA cells that received EVs derived macrophages. The SVGA cells (0.1 million cells/well/12well plate) were treated with EVs derived from control (DMSO) and CSC-exposed (10 µg/ml/day for 7 days) U937 macrophages once daily for 3 days. After 3 days of treatments, Western blot was performed with an equal amount of protein loaded (5 µg of protein) (**a**) to measure the expression of CYP2A6 (**b**), SOD1 (**c**), and catalase (**d**). One-way ANOVA with Tukey’s multiple comparison test was used to find the significance among the groups. **CEV** represents EVs isolated from the control group; **CSCEV** represents EVs isolated from the CSC-treatment group. *, **, and *** represent *p* < 0.05, *p* ≤ 0.01, and *p* ≤ 0.001 respectively when compared to control; #, ##, and ### represents *p* < 0.05, *p* ≤ 0.01, and *p* ≤ 0.001 respectively when compared to CSC.

We have earlier shown that nicotine alone and CSC (major tobacco constituents) induce oxidative stress through cytochrome P450 (CYP) 2A6-mediated pathway in U937 macrophages^38,48^. We have recently demonstrated that exposure to nicotine in rats increases the packaging of CYP2A6 and IL-1β in their plasma EVs^47^. Therefore, we intend to test whether the increased levels of IL-1β are associated with the CY2A6-induced oxidative stress pathway in SVGA cells. We observed that the protein expression of CYP2A6 (Fig. 4a,b; Supplementary Fig.S3) and antioxidant enzymes (SOD1 and catalase; Fig. 4a,c and d; Supplementary Fig. S3) did not vary in the groups that received EVs derived from control or CSC-exposed macrophages. The levels of CYP2A6 and AOEs also did not change when the SVGA cells were further exposed to CSC. However, as expected, the expressions of CYP2A6 and one of the AOEs, catalase, were significantly increased in SVGA cells upon CSC exposure alone. Though not statistically significant, addition of CEV and CSCEV in the presence of CSC appear to decrease CYP2A6 and catalase. These results suggest that the expression of IL-1β in astrocytes by macrophage-derived EVs is not likely associated with the CYP2A6-mediated oxidative stress pathway.

### Extracellular vesicles derived from HIV-infected U1 macrophages also enhance IL-1β, but not CYP2A6 and antioxidant enzymes in SVGA cells

**Figure 5:**
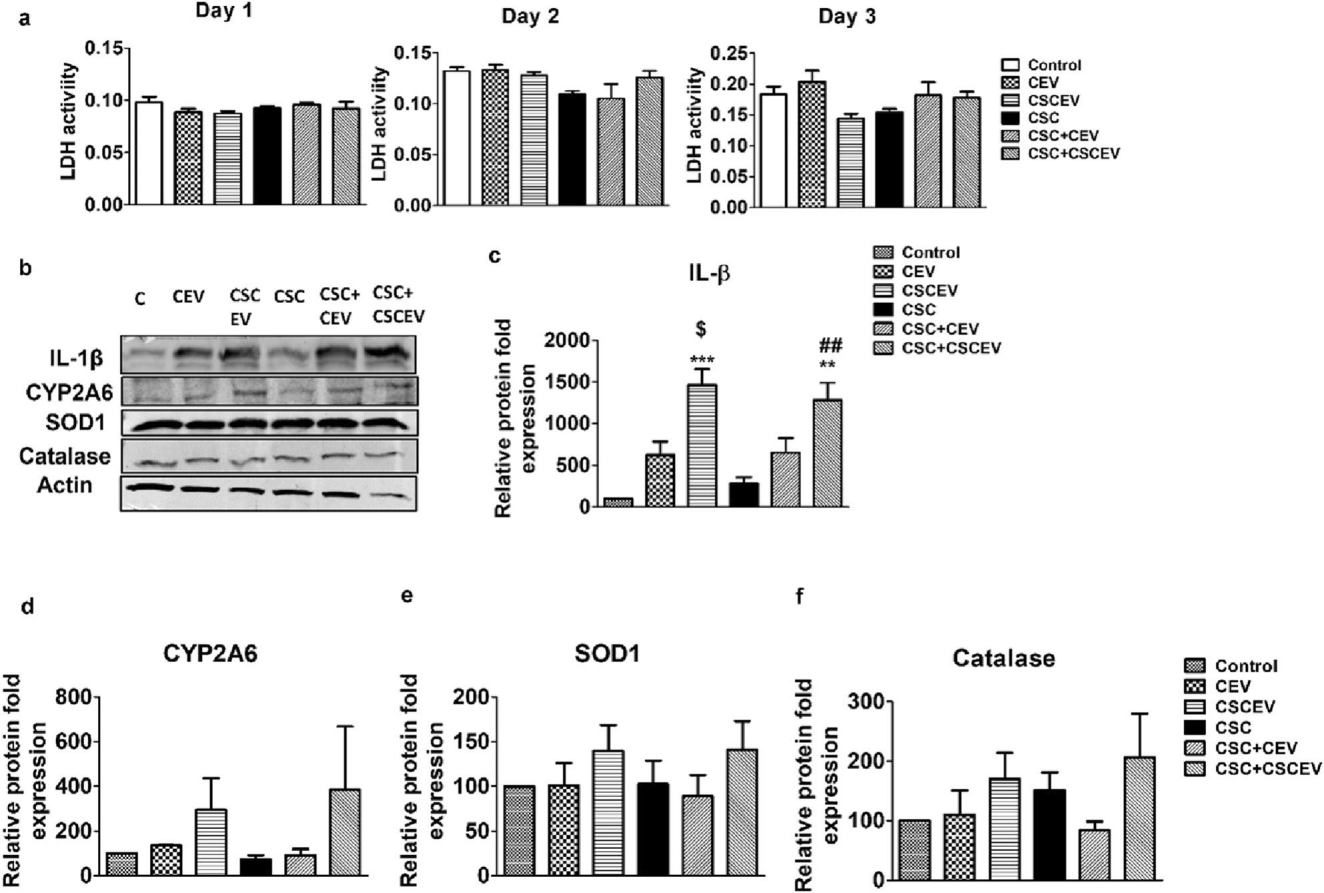
Effect of EVs derived from HIV-infected U1 macrophages on cytotoxicity and levels of IL-1β, CYP2A6, and AOEs in SVGA cells. The SVGA cells (0.1 million cells/well/12well plate) were treated with EVs-derived from control (DMSO) and CSC-exposed (10 µg/ml/day for 7 days) U1-macrophages once daily for 3 days in the presence and absence of CSC (10 µg/ml/day). After 3 days of treatments, LDH levels were measured (**a**). Western blot was performed with an equal amount of protein loaded (5 µg of protein) to measure the protein expression of IL-1β (b&c), CYP2A6 (**b** & **d**), and antioxidant enzymes (SOD1 and Catalase) (**b**, **e** & **f**)). One way ANOVA with Tukey’s multiple comparison test was used to find the significance among the groups. CEV represents EVs isolated from the control group; CSCEV represents EVs isolated from the CSC-treatment group. *, **and *** represent *p* < 0.05, *p* ≤ 0.01, and *p* ≤ 0.001 respectively when compared to control; #, ##and ### rep-resents *p* < 0.05, *p* ≤ 0.01, and *p* ≤ 0.001 respectively when compared to CSC.

As described above, we have also shown differential modulation of various cytokines, including IL-1 $\beta$ in HIV-infected U1 macrophage-derived EVs in the absence and presence of CSC^20^. Further, IL-1β is one of the most important proinflammatory cytokines, modulated in both macrophages and astrocytes upon HIV infection, and subsequently, cause HIV pathogenesis, including neuroinflammation^46,49–51^. Therefore, we wanted to examine whether EVs derived from HIV-infected macrophages when exposed to astrocytes also, or even further, enhance the levels of IL-1β in astrocytes. To explore this, we repeated the experiments as mentioned above (Figs. 2, 3, 4) with U1-derived EVs in the absence and presence of CSC. We observed no significant toxicity after 3 days of treatment (Fig. 5a). As seen with U937-derived EVs, IL-1β levels were increased in the SVGA cells that received EVs compared to the respective controls. The IL-1β levels were further increased in the SVGA cells, which received EVs from CSC-treated U1 macrophages (CSCEV) compared to EVs from control-treated U1 macrophages (CEV). (Fig. 5b and c; Supplementary Fig. S4). There was no further increase in the IL-1β levels when SVGA cells were treated with CSC. Although CYP2A6 and AOEs were slightly elevated in SVGA cells when exposed to EVs derived from U1 cells (Fig. 5b,d–f; Supplementary Fig. S4), it was not statistically significant. Overall, these results further verify the role of EVs in cell–cell communication, not only from HIV-uninfected but also from HIV-infected cells to astrocytes.

### Extracellular vesicles derived from HIV-uninfected U937 and HIV-infected U1 macrophages enhance IL-1β levels in neuronal cells

**Figure 6:**
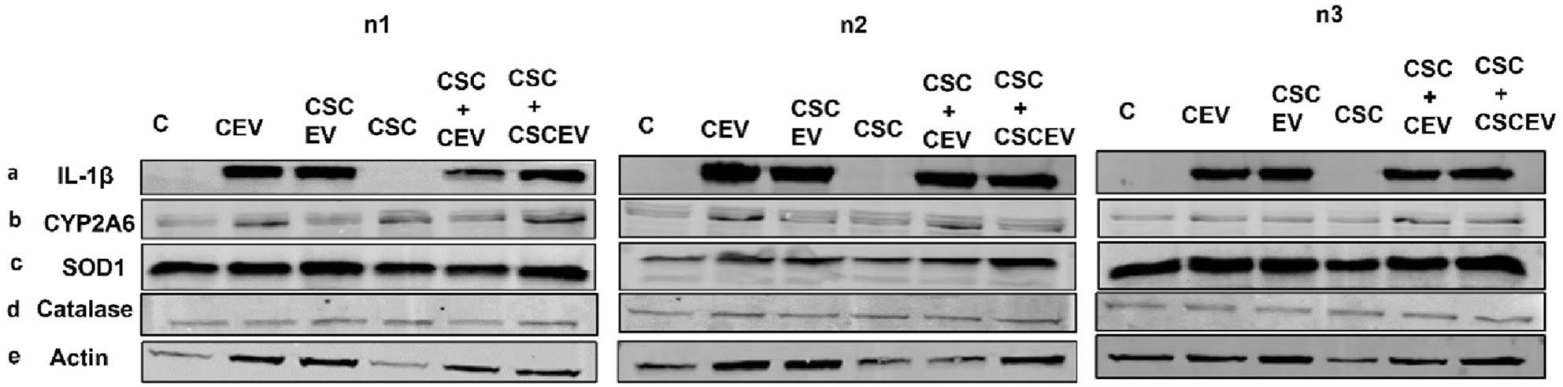
Effect of EVs derived from uninfected macrophages on the levels of IL-1β, CYP2A6, and AOEs in SHSY-Y5 neuronal cells. The neuronal cells (0.1 million cells/well/12well plate) were treated with EVs-derived from control (DMSO) and CSC-exposed (10 µg/ml/day for 7 days) U937 macrophages once daily for 2 days in the presence and absence of CSC (10 µg/ml/day). We observed more than 50% cell death after 2 days of treatment. Therefore, we harvested the cells after 2 days of treatment. We performed a Western blot with an equal amount of protein loaded (5 µg of protein) to measure the protein expression of IL-1β (**a**), CYP2A6 (**b**), and antioxidant enzymes (**c** & **d**). ‘**n**’ represents number of replicates. **CEV** represents EVs isolated from the control group; **CSCEV** represents EVs isolated from the CSC-treatment group.

**Figure 7:**
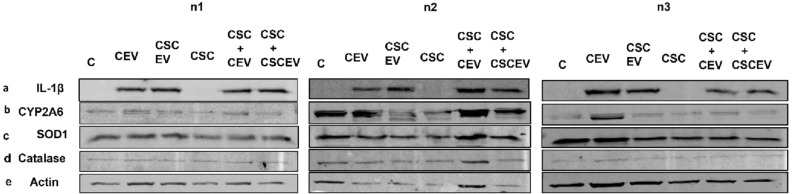
Effect of EVs derived from HIV-infected U1 macrophages on the levels of IL-1β, CYP2A6, and AOEs in SHSY-5Y cells. The neuronal cells (0.1 million cells/well/12well plate) were treated with EVs-derived from control (DMSO) and CSC-exposed (10 µg/ml/day for 7 days) U1-macrophages once daily for 2 days in the presence and absence of CSC (10 µg/ml/day). We observed more than 50% cell death after 2 days of treatment. Therefore, we harvested the cells after 2 days of treatment. We performed Western blot with an equal amount of protein loaded (5 µg of protein) to measure the protein expression of IL-1β (**a**), CYP2A6 (**b**), and antioxidant enzymes (**c** & d). ‘**n**’ represents the number of replicates. **CEV** represents EVs isolated from the control group; **CSCEV** represents EVs isolated from the CSC-treatment group.

As described in the introduction section, oxidative and inflammatory factors released from HIV-infected macrophages and microglia eventually interact with neurons causing neuronal damage leading to HIV-associated neurocognitive disorders (HAND)^9,15,52^. Although EVs have been implicated in these interactions, there is no experimental evidence that EVs carry these factors from macrophages and deliver them to neuronal cells in HIV-infected or uninfected models. Therefore, we intend to test whether the EVs derived from macrophages can transfer IL-1β to the neuronal cells. To test this, we exposed differentiated SHSY-5Y neuronal cells with EVs derived from both HIV-uninfected U937 and infected U1 macrophages (Figs. 6 and 7, respectively; Supplementary Fig. S5 & S6 respectively). We observed that EVs derived from U937 and U1 macrophages transfer IL-1β to neuronal cells (Figs. 6a and 7a, respectively).

Interestingly, we observed that the basal IL-1β protein expression in both control and CSC-treated neuronal cells was absent (Figs. 6a and 7a). Therefore, we could not quantify and compare the relative protein expression of IL-1β levels with other study groups. Similar to the results observed in SVGA cells, we also did not observe any significant change in CYP2A6 or AOEs in SHSY-5Y neuronal cells when exposed to EVs derived from both U937 and U1 macrophage. As expected and similar to the astrocyte results (Fig. 4b,d and 5b,f), there was an increase in CYP2A6 and catalase in the neuronal cells by CSC exposure alone (Figs. 6b, d and 7b, d). It can be noted that the housekeeping protein, actin, also changed in SH-SY5Y cells when they were exposed to EVs derived from U1 cells, especially in the absence of CSC. Nevertheless, the levels of CYP2A6 and AOEs were largely unchanged.

## Discussion

Our recent findings with the EVs derived from uninfected (U937), and HIV-infected (U1) macrophages have shown the effect of CSC exposure on differential packaging of various cytokines and AOEs in EVs^20^. The current study extends our work to establish the role of EVs derived from CSC-exposed uninfected and infected macrophages in cellular communication among astrocytes and neuronal cells. The cell–cell communication between the cells of myeloid origin (macrophages and microglia), astrocytes, and neurons is very important, particularly in context to HIV neuropathogenesis. We observed that the HIV-uninfected and HIV-infected macrophages package inflammatory cytokine IL-1β in EVs. When exposed to astrocytic and neuronal cells, the EVs derived from U937 and U1 cells transfer IL-1β to astrocytic and neuronal cells and enhance their cellular levels. Compared to control, CSC exposure to macrophages perhaps packages more IL-1β in EVs, and upon transfer to astrocytes and neuronal cells, further increases the levels of IL-1β in these cells. This is the first evidence of cell–cell communication of macrophages with astrocytes and neurons via EVs containing IL-1β, especially in context to HIV neuropathogenesis.

HIV does not directly infect neurons, and neurological complications observed in AIDS have been attributed to indirect viral effects. Indeed, mounting data suggest that HIV-infected macrophages and microglia result in cellular activation and produce neurotoxins leading to neuronal damage^9,15,53^. Further, soluble factors released by astrocytes also contribute to neuronal damage in HIV patients^54^. A study by Pulliam et al. suggests that HIV-infected both in vitro and ex vivo (HIV derived from AIDS patients’ peripheral blood) macrophages produce soluble factors that cause reproducible structural and functional abnormalities in the human brain aggregates^55^. These soluble neurotoxic factors include cytokines, excitatory amino acids, eicosanoids, and reactive oxygen or nitrogen species.

Among neurotoxic factors, the role of cytokines is gaining importance in cellular communication as they are reported to be packaged in EVs^20,28,47,56–58^. The cargo packaged in EVs is protected from destruction and enzymatic degradation^59^. Since the cytokines are packaged in EVs, they can remain stable in the circulation and reach distant cells and cause inflammation. Further, EV-associated cytokines can serve as biomarkers of various disease conditions. Konadu et al. reported that most cytokines and chemokines are significantly higher in plasma EVs of HIV-infected subjects than their plasma and non-infected subjects^60^.

Further, the packaging of cytokines in EVs varies depending upon the type of exposure/stimuli. We reported a difference in cytokines levels packaged in EVs derived from the plasma of HIV-infected alcohol drinkers and cigarette smokers^57^. Previous studies in our group also showed that cytokines, CYPs, and AOEs could be packaged in EVs from HIV-infected and uninfected samples in vitro^20^ and in vivo^28^. Nevertheless, whether these EV-harboring inflammatory cytokines affect recipient cells via cell–cell communication has not been explored.

The role of EVs in cell–cell communication has been implicated in various diseases^10,19–22^. Previously, we have demonstrated the role of EVs in drug abuse-mediated HIV replication and cellular toxicity^41,61^. One study has shown that EVs from healthy human plasma increase alcohol and acetaminophen (APAP) mediated toxicity in hepatic cells and HIV-infected macrophages. We have also demonstrated that EVs derived from alcohol-exposed mice contain higher levels of CYP2E1 and lower levels of AOEs than control mice. Further EVs derived from alcohol-exposed mice cause significantly higher toxicity in HIV-infected cells than the EVs from the control group^61^. In another study, we have demonstrated that EVs from cervical cancer cells exacerbate viral replication in HIV-infected macrophages by transferring CYPs and human papillomavirus (HPV) oncoproteins^62^. More relevant to the current study, we have demonstrated that EVs from CSC-treated uninfected cells showed increased cytotoxicity and DNA damage in HIV-uninfected macrophages^41^. However, EVs from CSC-treated uninfected and infected cells showed a protective effect against cytotoxicity and viral replication in HIV-infected macrophages compared to the CSC treatment group^41^. In contrast, in the present study, EVs from both uninfected and infected macrophages did not alter SVGA cells' toxicity for at least 3 days. This suggests that the effect of EVs from macrophages in the recipient cells varies depending on the recipient cells' type and exposure time.

Our previous findings suggest that CSC exposure increases packaging of IL-β in EVs derived from macrophages, both in the absence and presence of HIV infection^20^. Further, we also observed that EVs derived from macrophages contain CYP2A6 and AOEs^20^. In the current study, for the first time, we demonstrated that inflammatory molecules packaged in macrophage derived EVs could be transferred to the brain cells and elevate the levels of inflammatory molecules in the recipient cells. We observed that CYP2A6 and AOEs packaged in EVs derived from macrophages can be transferred to astrocyte and neuronal cells, but only IL-1β levels were significantly increased in the recipient cells. This could be due to the high percentage of IL-β packaged in EVs derived from macrophages, as we have shown that the expression of IL-1β was much higher in EVs than their respective cells **(**Fig. 3c**)**. Our findings also corroborate with Fitzgerald's study, suggesting that the percentage of most of the cytokines produced by monocytes are more in the EV-associated form than in the free state^56^.

Further, our results also suggest that the increased expression of IL-1β in astrocytes and neuronal cells by macrophage-derived EVs is not associated with the CYP2A6-mediated oxidative stress pathway. The synthesis and release of IL-1β from EVs are highly regulated by activating the NLRP3 inflammasome pathway, which may explain the increase in IL-1β levels^63^ in the brain cells, and independent of the CYP2A6-mediated oxidative stress pathway. Inflammation is a significant hallmark of HIV infection and the pro-inflammatory cytokine, IL-1β, plays an important role in inducing neuronal injury and death in HIV subjects^64,65^. Further, the mRNA expression of IL-1β was higher in HIV-infected patients with dementia than in those without dementia^66^. In addition, IL-1β stimulates astrocytosis^67–69^, which has been implicated in various neurological disorders^70–72^, including HIV-associated neuropathogenesis^54,72,73^. In the present study, the EVs derived from macrophages increased astrocyte IL-1β levels that could lead to astrocytosis and neuronal damage/death.

The IL-1β exacerbates HIV and its associated complications^46,74–76^. It is well known that HIV infections induce IL-1β production. The produced IL-1β, in turn, exacerbates HIV pathogenesis by various mechanisms. One of the possible mechanisms by which IL-1β contributes to neuronal damage is by increasing the infiltration of HIV-infected monocytes into the brain^74,77^. The infiltrated HIV-infected monocytes are one source for entry of infectious virus particles into the brain. HIV does not directly infect the neurons but can infect resident macrophages/microglia and astrocytes. These infected cells produce neurotoxins, including IL-1β, packaged in EVs, released into circulation, and transferred to neuronal cells. Reduction in neuronal apoptosis/death with IL-1β inhibition strengthens the role of IL-1β in neuroinflammation/damage^78,79^.

### In conclusion, the current study demonstrates the cell–cell interaction between macrophages and brain cells mediated via EVs

The increased IL-1β levels via EV-mediated cell–cell interactions could contribute to neuroinflammation in HIV-uninfected and HIV-infected settings. Although this is an important finding about the role of EVs in the interaction between macrophages and brain cells, astrocytes and neurons, we will validate this using in vivo wild-type and HIV mice models in future studies. Further, we also plan to study whether the EVs derived from peripheral macrophages can cross the BBB and deliver the IL-1β to the brain cells using in vitro BBB models and in vivo animal models.

## Materials and methods

### Cell culture and treatment

Human neuroblastoma cells, SH-SY5Y and human astrocytic cell line SVGA were obtained from American Type Culture Collection (ATCC, Manassas, VA). The EVs were isolated from the culture media of monocyte-derived macrophages. The chronically infected U1 cells (NIH AIDS Reagent Program, Germantown, MD) and U937 cells (ATCC, Manassas, VA) were differentiated into macrophages. Our group and many other researchers have extensively utilized model cell lines U937 and U1 to explore the effects of drug abuse in HIV infection^38,40,41,80^. U1 is chronically infected HIV promonocytic U937 cells. Earlier studies performed with these cell lines have been repeatedly confirmed and found to be strongly correlated with primary macrophages^9,81^. The cells were maintained and differentiated as described in one of our pulications^20^. We have previously demonstrated that treating U937 and U1 cell lines with CSC did not show any toxicity up to 6–8 days^20^, and showed increased packaging of IL-1β in EVs. Therefore, we treated the cells with 10 μg/ml of cigarette smoke condensate (CSC) (Murty Pharmaceuticals, Inc, KY) once daily for 7 days (long-term exposure). At the end of the treatment, cells were harvested, and the supernatant was collected for EV isolation.

**The human SVGA astrocytic cell lines** were cultured in DMEM containing 10% (v/v) FBS, 1% of 40 U/ml penicillin, and 40 mg/ml streptomycin at 37 °C with 5% CO2. The SVGA cells were treated with EVs derived from long-term CSC-exposed (10 μg/ml once daily for 7 days) U937 and U1 differentiated macrophages in the presence and absence of CSC for 3 days.

**The human SH-SY5Y neuroblastoma cell lines** were maintained and differentiated as described previously with minor modifications^82^. They were cultured in MEM + F12 media (1:1) containing 10% (v/v) FBS, 1% NEAA, 1% of 40 U/ml penicillin, and 40 mg/ml streptomycin at 37 °C with 5% CO2. For differentiation into neuron-like type, 0.25 million cells/well were seeded in a 12 well plate with 1 ml of the media and incubated overnight at 37 °C with 5% CO_2_. The next day, the media was removed, and fresh media containing 10 μM all-trans retinoic acid was added to the cells and incubated for 3 days. On day 4, the media was removed, and cells were washed with serum-free media (MEM + F12 media). After washing, 1 ml of serum-free media (MEM + F12 media (1:1) supplemented with 1% NEAA and 1% of 40U/ml penicillin and streptomycin) containing BDNF (50 ng/ml) was added to the cells and allowed them 3 more days to differentiate. On day 7, the media was removed, and cells were washed and added with serum-free media (MEM + F12 media). These differentiated cells were treated with EVs derived from long-term CSC-exposed (10 μg/ml once daily for 7 days) U937 and U1 differentiated macrophages with and without CSC for 2 days.

### EV isolation and characterizations

EVs were isolated from supernatant of U937 and U1 cell culture media using Invitrogen-Total Exosome Isolation (from cell culture media) kit (Life Technologies, NY) as described earlier20,41. Briefly, the cells and debris from the media were removed by centrifugation at 2000 *g* for 30 min. After centrifugation, the resultant supernatant containing the cell-free culture media was mixed with 0.5 volume of the Total Exosome Isolation (from cell culture media) reagent. The cell culture media/reagent mixture was vortexed until there was a homogenous solution and incubated overnight at 4 °C. The mixture was centrifuged at 10,000 *g* for 1 h at 4 °C at the end of incubation. The protein concentration of EV samples were quantified using the Pierce BCA Protein Assay Kit (Thermo Scientific). We also determined the presence of EV marker proteins CD63 and Alix using Western blot analysis as described below. Acetylcholinesterase (AChE) activity was measured using the fluorescent Amplex® Red Acetylcholine/Acetylcholinesterase Assay Kit (Molecular Probes, Invitrogen) as previous reported^47^. Size and Zeta potential of EVs were measured using the Zetasizer Nano-ZS (Malvern Instruments Inc, Malvern, UK) as described earlier^41^. In brief, EV pellets were resuspended in 800 µl 0.22 µm filtered 1XPBS before being subjected to dynamic light scattering in the Zetasizer. Each sample was scanned for three times for EV size and Zeta potential measurement.

### Cytokine analysis

The levels of cytokines (pro-inflammatory: TNF-α, IL-1β, IL-6, IL-8, IL-18; anti-inflammatory: IL-1RA, IL-10) and chemokines (MCP-1 and RANTES) were measured in the cell culture supernatant of SVGA cells using customized human 9-Plex ProcartaPlexTM multiplex immunoassay (ThermoFisher Scientific, Waltham, MA) as per previously described method^83^. Briefly, SVGA cells were treated with CSC (10 μg/ml) and, in combination with EVs obtained from long-term CSC, exposed U937 differentiated macrophages. According to the manufacturer’s protocol, in a 96-well plate, the standards, samples, and magnetic beads were added and mixed using a plate shaker for 1 h at room temperature and subsequently incubated overnight at 4 °C. The wells were washed at the end of incubation, and subsequent addition of streptavidin-PE and detection antibody was followed by reagent wash at all steps. The quantification of cytokines was performed as per the manufacturer’s protocol. The Magpix system was used to measure the concentration of cytokines and chemokines. xPONENT version 3.1 software (https://www.luminexcorp.com/xponent/#overview) was used to analyze the data.

### LDH activity

The cytotoxic effect of EVs derived from U937 and U1 macrophages in SVGA cells was determined by LDH estimation with CyQUANT LDH Cytotoxicity Assay Kit (Catalog no. C20300, ThermoFisher Scientific) following the manufacturer’s protocol.

Briefly, the SVGA cells were treated with EVs in the presence and absence of CSC for 3 days. Fifty microliter of cell culture supernatant was collected on each day of treatment and replaced with an equal amount of fresh media. The collected 50 µl media was transferred to a 96-well plate, and 50 µl of LDH reaction mixture was added to the media samples and incubated at room temperature for 30 min. Cytotoxicity was determined with Pierce LDH Cytotoxicity Assay Kit (ThermoFisher Scientific, Grand Island, NY, USA) in the cell culture supernatant collected from differentiated U1 macrophage media following the manufacturer’s instructions. Briefly, 50 μl of the LDH reaction mixture was mixed with 50 μl of the culture supernatant in a 96-well plate followed by 30 min incubation at room temperature. At the end of incubation, 50 μl of LDH stop solution was added to stop the reaction. Background absorbance at 680 nm was subtracted from absorbance at 490 nm. A higher absorbance suggests higher toxicity.

### Western blotting

The cell lysis was performed with RIPA buffer, followed by protein quantification using Pierce BCA Protein Assay Kit (Thermo Scientific). A aliquot of 5–10 µg protein from cell extracts were loaded onto 10% SDS-PAGE gel to determine the protein expression, and the next steps were performed as described earlier^84^. We evaluated the protein expression of pro-inflammatory cytokine (IL-1β), proteins associated with antioxidant enzymes (SOD1 and CAT), and CYP2A6. Upon running the gel, proteins were transferred onto PVDF membrane and blocked in Li-Cor blocking buffer (LI-COR Biosciences, Lincoln, NE) for 1 h. After blocking, membranes were incubated overnight at 4 °C with primary antibodies; CD63 rabbit mAb (1:500 dilution, Proteintech), Alix (1:700 dilution, Proteintech), SOD1 mouse mAb (1:500 dilution, Santa Cruz Biotechnology), catalase mouse mAb (1:500 dilution, Santa Cruz Biotechnology), IL-1β rabbit pAb (1:500 dilution, Proteintech), CYP2A6 rabbit mAb (1:500 dilution, Proteintech), and β-Actin mouse mAb (1:2000 dilution, Cell Signaling). The membranes were washed the next day and incubated with either goat anti-mouse or goat anti-rabbit secondary antibody (1:10,000 dilution, LI-COR Biosciences) for 1 h at room temperature. At the end of incubation, the membranes were washed, and the signal was detected using the LI-COR (Biosciences, Lincoln, NE). LI-COR Image Studio Lite version 4.0. (Nebraska, USA) software was used to perform the densitometry analyses of the proteins.

### Statistical analysis

The data of all experiments are represented as mean ± SEM of at least three independent experiments. One-way ANOVA followed by Tukey’s multiple comparisons test was performed using GraphPad Prism version 5.0.0 for Windows, GraphPad Software, San Diego, California USA, www.graphpad.com. The level of significance was set at *p* ≤ 0.05.

## Supplementary Information

Supplementary Information.

## Data Availability

All data generated or analyzed during this study are included in this published article (and its Supplementary Information files).
