# Supplementary material for: Extracellular vesicles released from macrophages modulates interleukin-1β in astrocytic and neuronal cells
Source: Sci Rep. 2023 Feb 21;13:3005. doi: 10.1038/s41598-023-29746-y (PMC9944928; doi:10.1038/s41598-023-29746-y)
Supplement: Supplementary file 1 — Supplementary Information. [file 41598_2023_29746_MOESM1_ESM.docx]

Title: Extracellular Vesicles Released from Macrophages Modulates Interleukin-1β in Astrocytic and Neuronal Cells.

**Sunitha Kodidela^1^, Namita Sinha^1^, Asit Kumar ^1^, Lina Zhou^1^, Sandip Godse^1^, and Santosh Kumar ^1*^**

^1^ Department of Pharmaceutical Sciences, College of Pharmacy, The University of Tennessee Health Science Center, Memphis, TN 38163, USA.

***Corresponding author:** Santosh Kumar, [ksantosh@uthsc.edu](mailto:ksantosh@uthsc.edu)

**Supplementary Figure S1a: Figure 1b original blots**

 Characterization of EVs using representative EV samples derived from U937 and U1 cells with and without CSC treatment. EVs were characterized using markers, CD63. CEV represents EVs isolated from the control group; CSC represents EVs isolated from the CSC-treatment group.


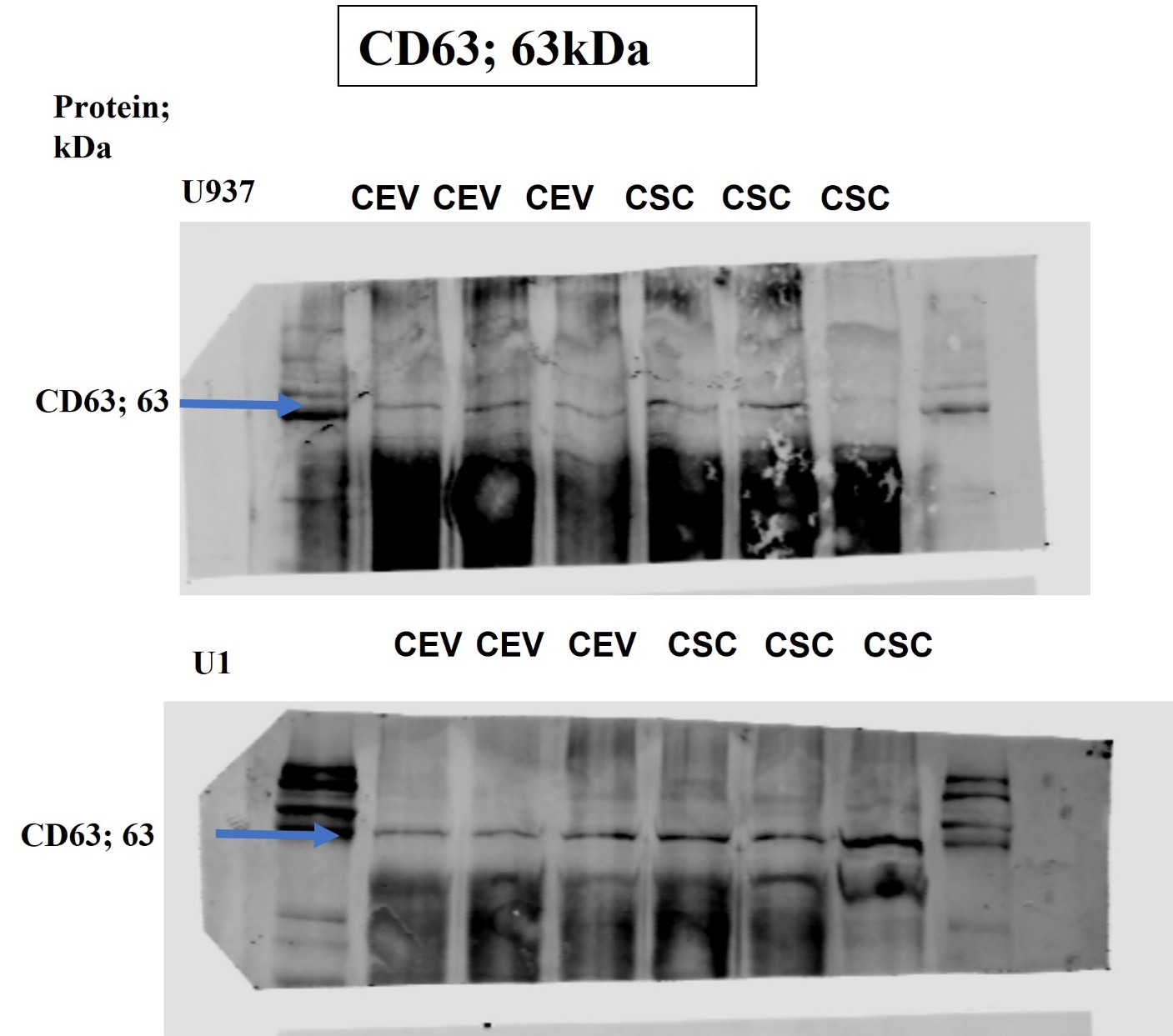


**Supplementary Figure S1b: Figure 1c original blots**

 Characterization of EVs using representative EV samples derived from U937 and U1 cells with and without CSC treatment. EVs were characterized using markers s, Alix. CEV represents EVs isolated from the control group; CSC represents EVs isolated from the CSC-treatment group.


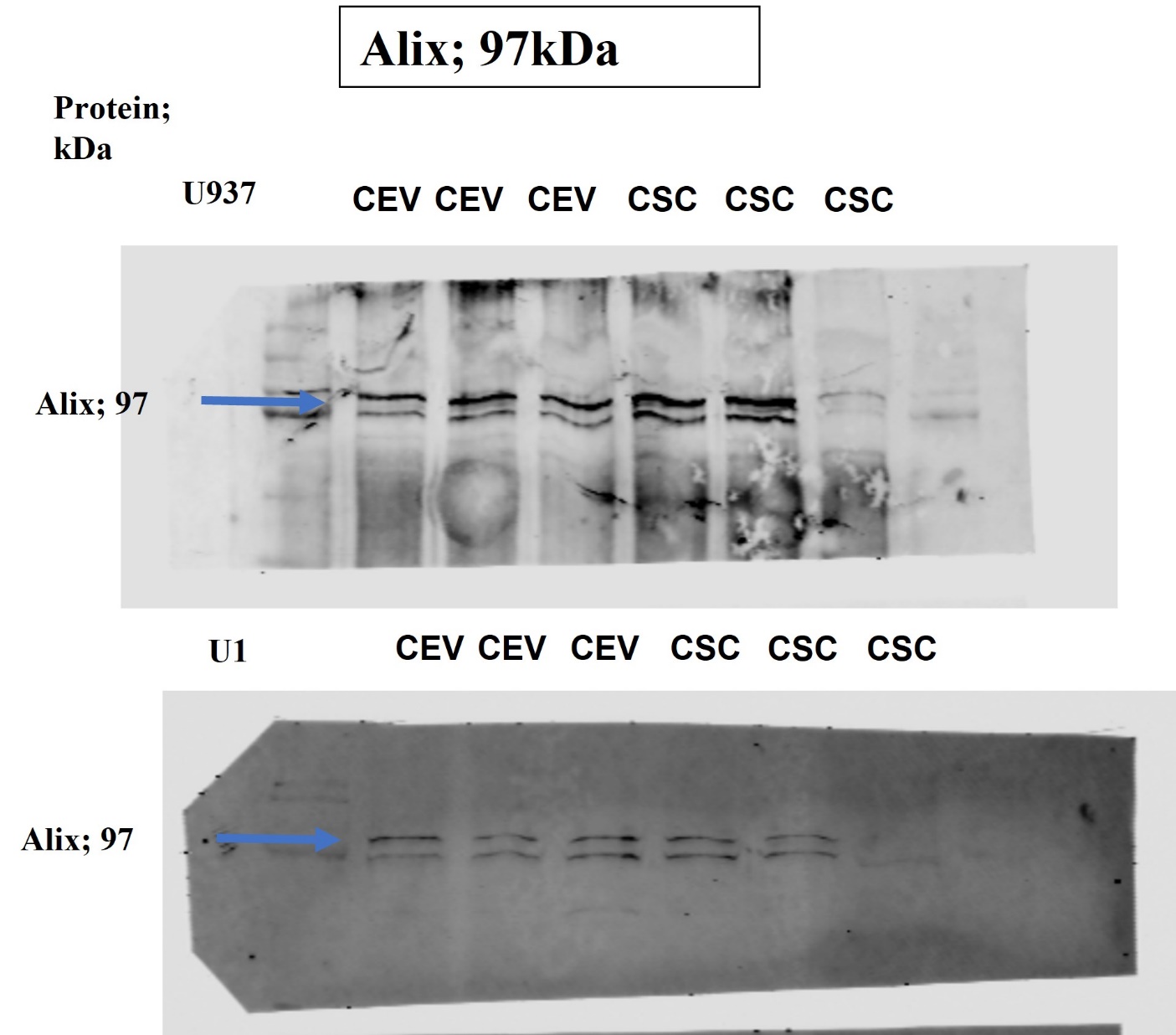


**Supplementary Figure S2: Figure 3 original blots a & c**

Effect of EVs derived from HIV-uninfected U937 macrophage on IL-1β protein expression in SVGA cells and comparison of IL-1β levels between EVs derived from U937 macrophages and their respective cells

CE represents EVs isolated from the control group; CSCEV represents EVs isolated from the CSC-treatment group; C-cell represents control U937 cells; CSC-cell represents CSC treated U937 cells.


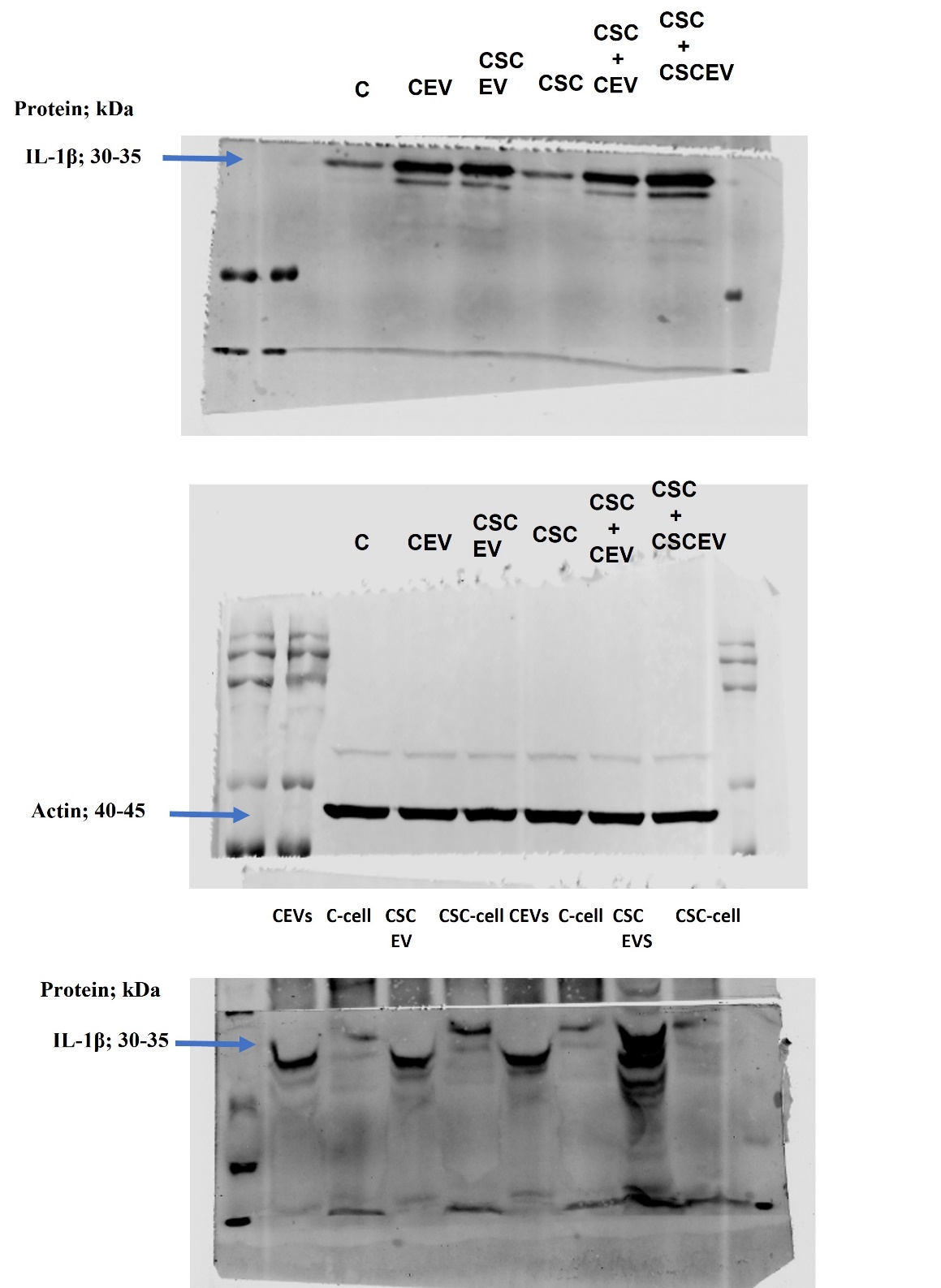


**Supplementary Figure S3: Figure 4 original blots (a):** Expression of CYP2A6 and antioxidant enzymes in SVGA cells that received EVs derived from macrophages

CEV represents EVs isolated from the control group; CSCEV represents EVs isolated from the CSC-treatment group.


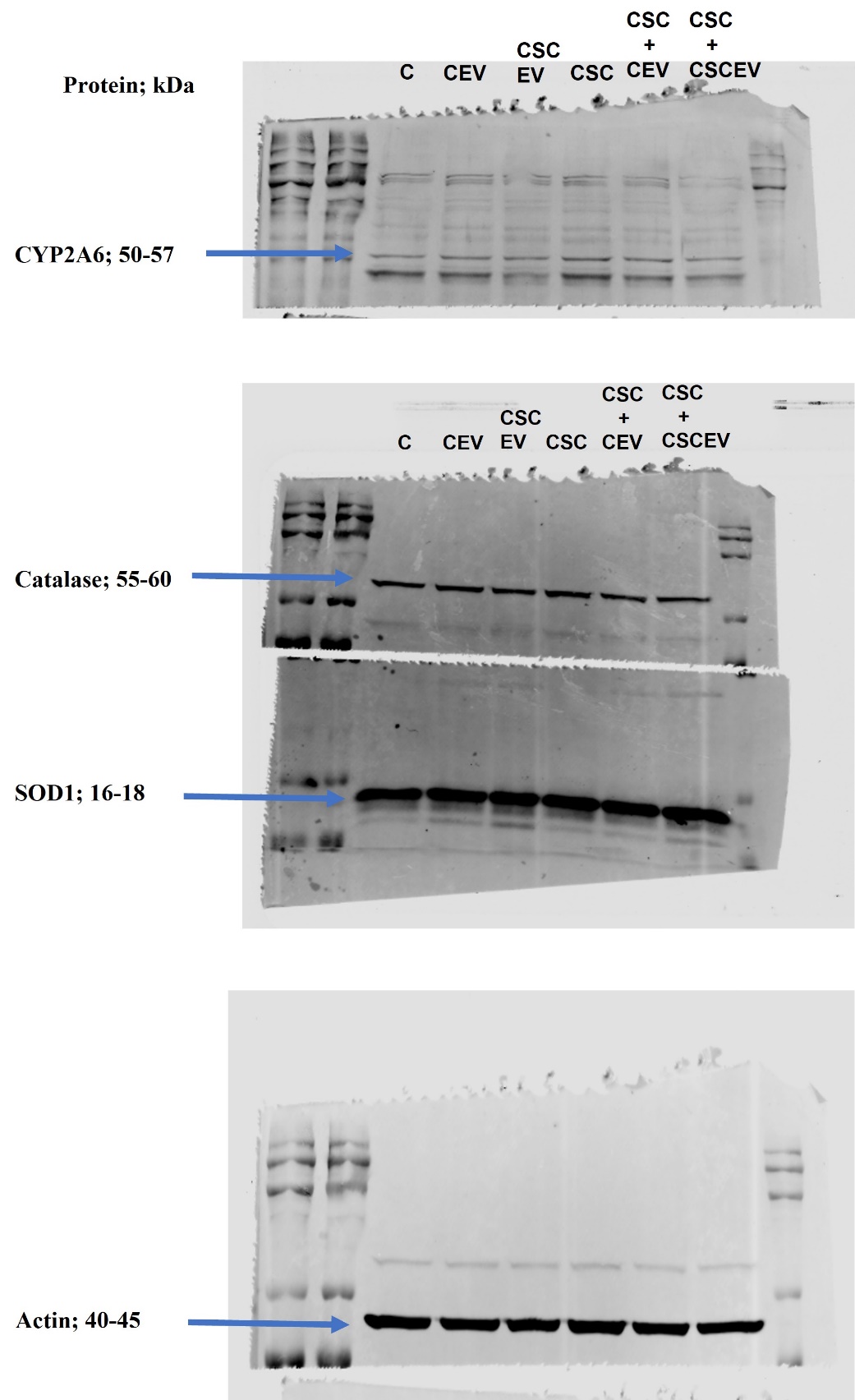


**Supplementary Figure S4 :** **Figure 5 original blots** : Effect of EVs derived from HIV-infected U1 macrophages on cytotoxicity and levels of IL-1β, CYP2A6, and AOEs in SVGA cells. CEV represents EVs isolated from the control group; CSCEV represents EVs isolated from the CSC-treatment group.

**Actin; 40-45**


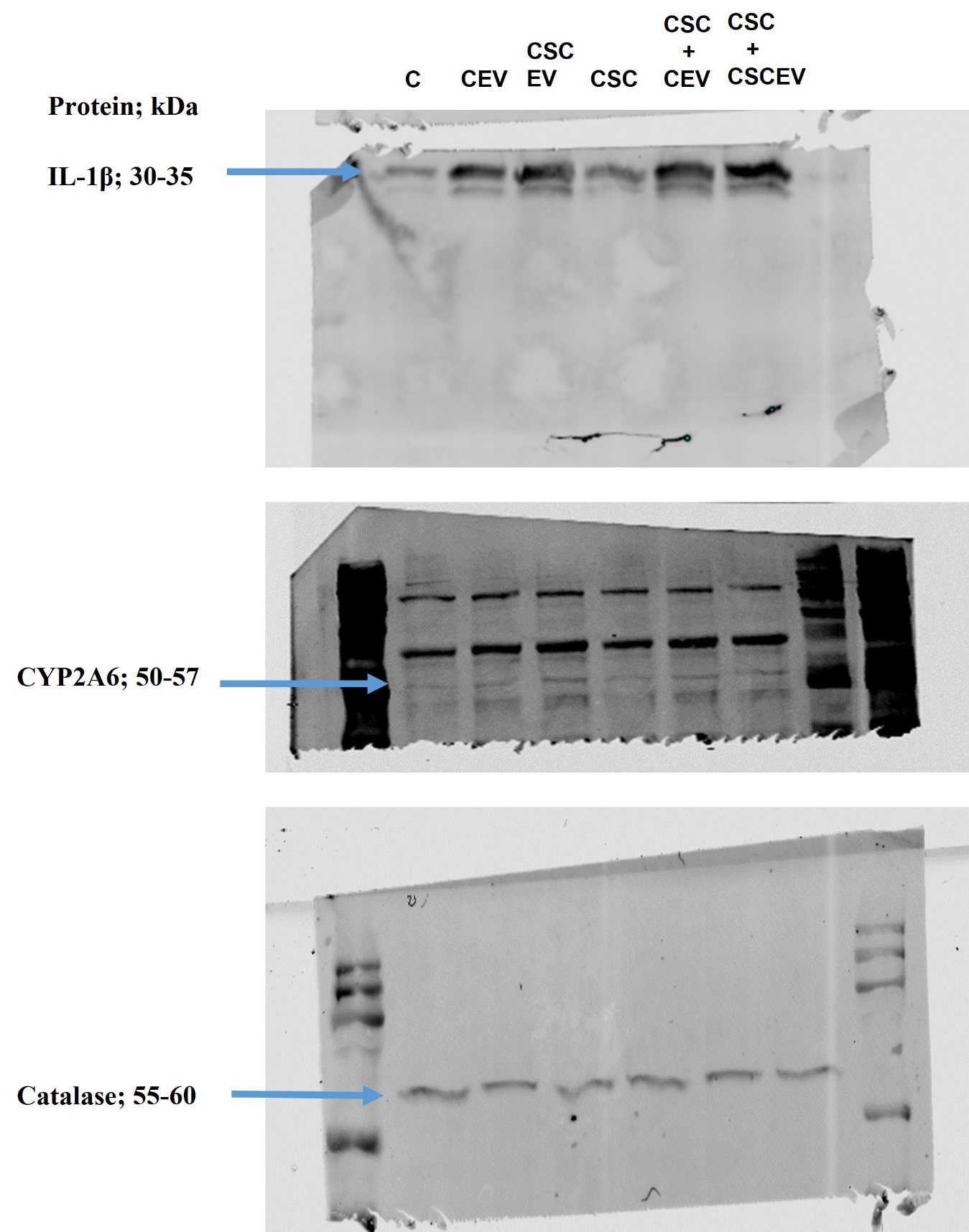


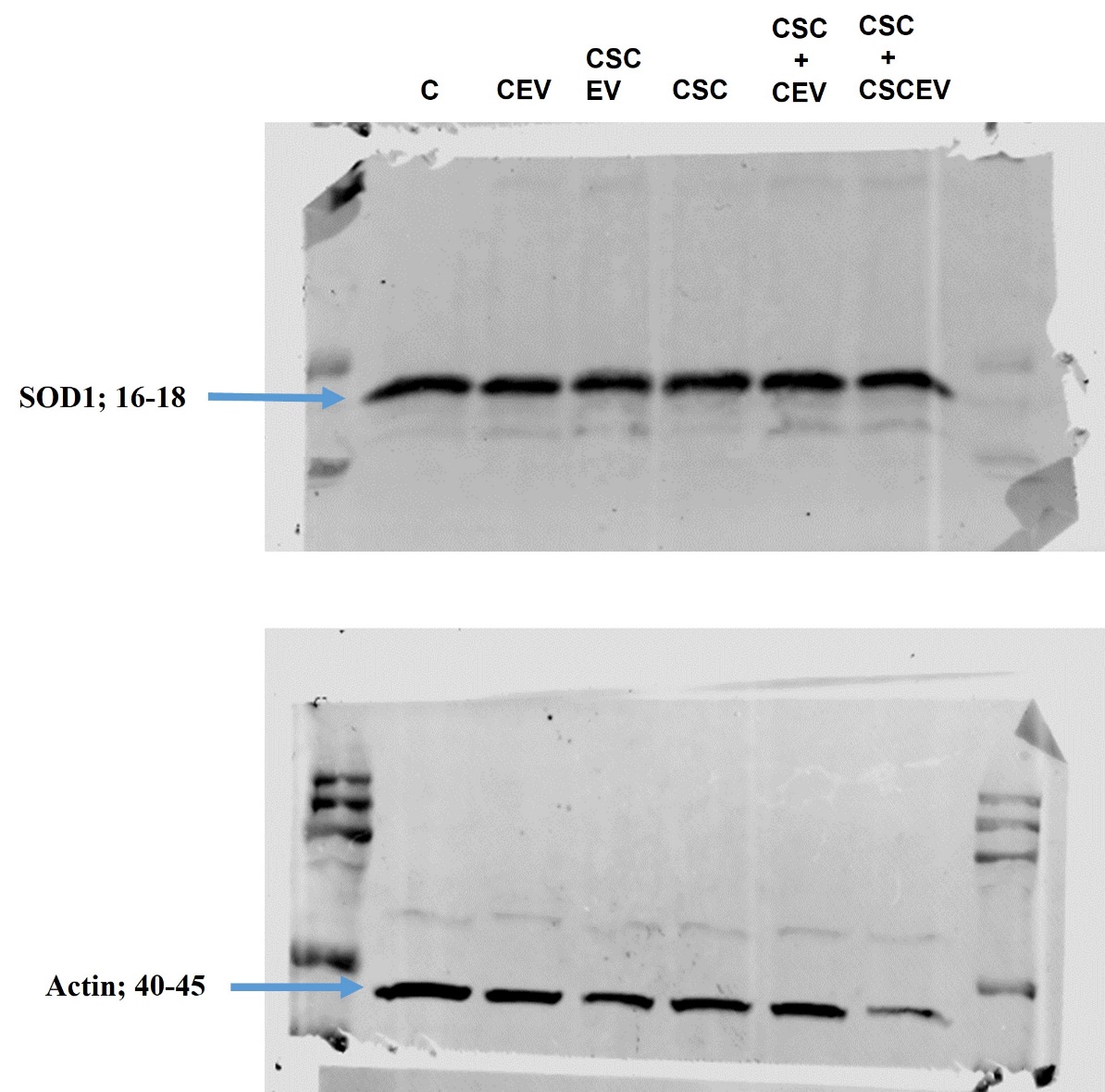


**Supplementary Figure S5:** **Figure 6 original blots**: Effect of EVs derived from uninfected macrophages on the levels of **IL-1β** in SHSY-Y5 neuronal cells. ‘n’ represents the number of replicates. CEV represents EVs isolated from the control group; CSCEV represents EVs isolated from the CSC-treatment group


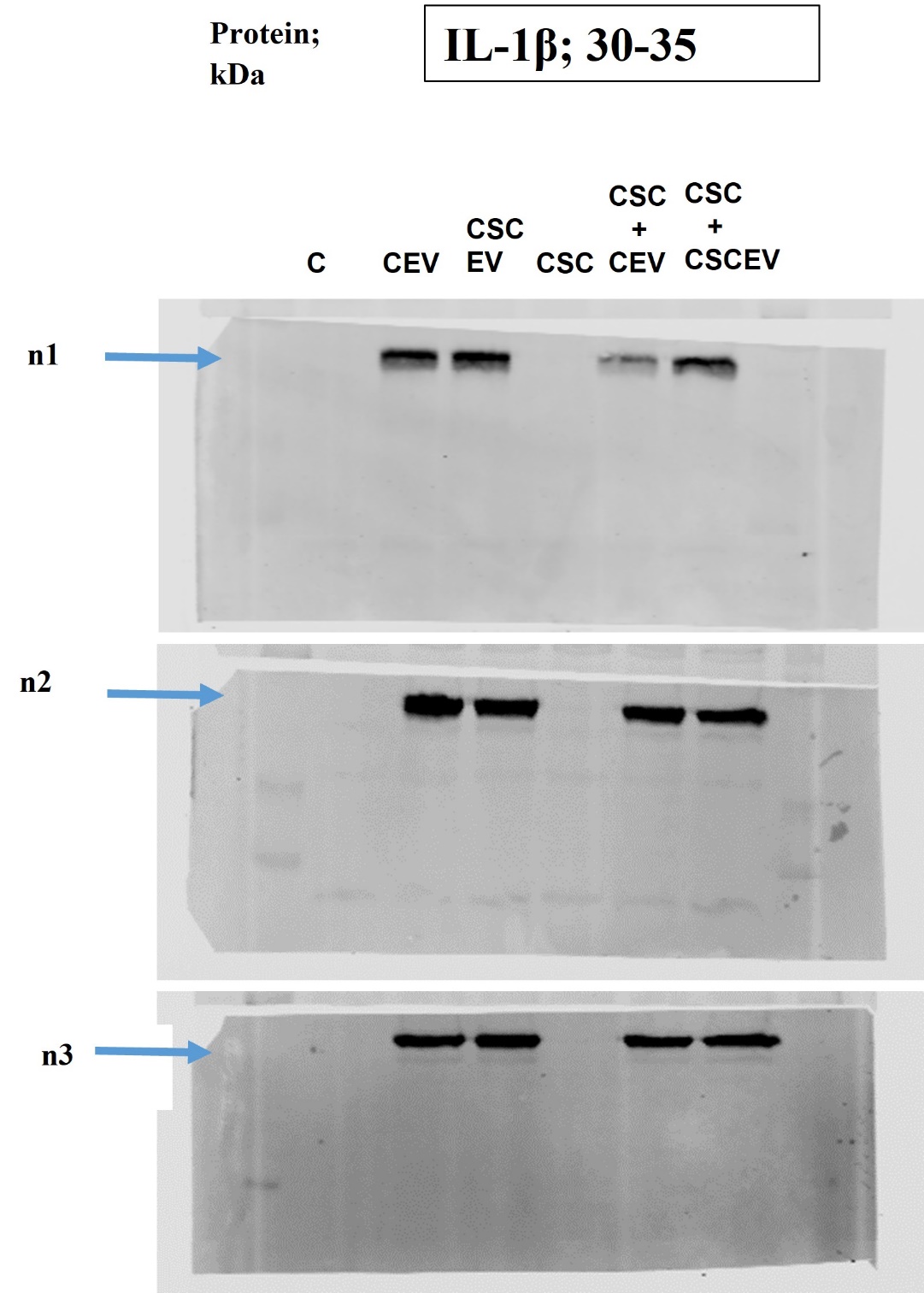


**Supplementary Figure S5:** **Figure 6 original blots**: Effect of EVs derived from uninfected macrophages on the levels of **CYP2A6** in SHSY-Y5 neuronal cells. ‘n’ represents the number of replicates. CEV represents EVs isolated from the control group; CSCEV represents EVs isolated from the CSC-treatment group


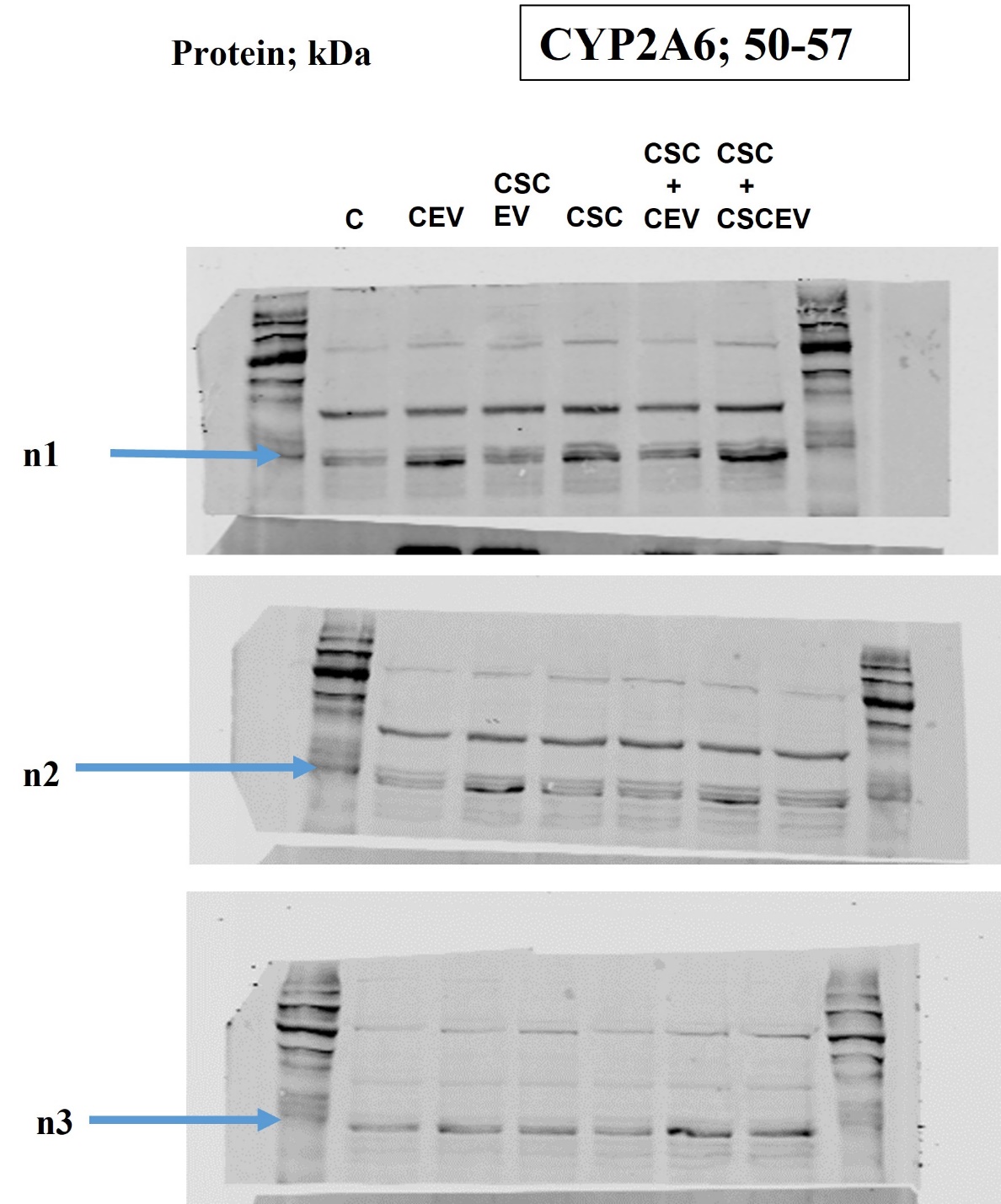


**Supplementary Figure S5:** **Figure 6 original blots**: Effect of EVs derived from uninfected macrophages on the levels of **catalase** in SHSY-Y5 neuronal cells. ‘n’ represents the number of replicates. CEV represents EVs isolated from the control group; CSCEV represents EVs isolated from the CSC-treatment group


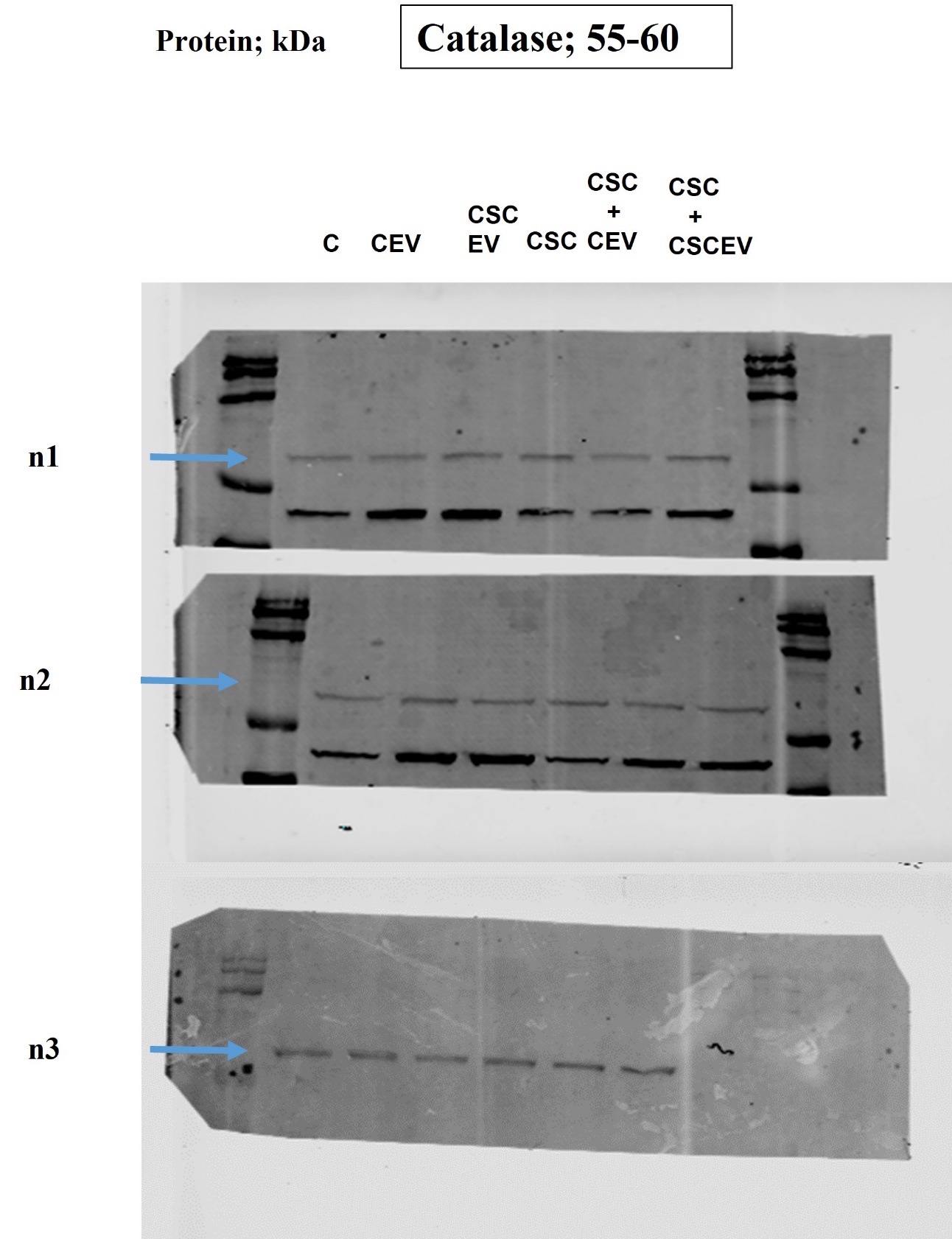


**Supplementary Figure S5:** **Figure 6 original blots**: Effect of EVs derived from uninfected macrophages on the levels of **SOD1** in SHSY-Y5 neuronal cells. ‘n’ represents the number of replicates. CEV represents EVs isolated from the control group; CSCEV represents EVs isolated from the CSC-treatment group


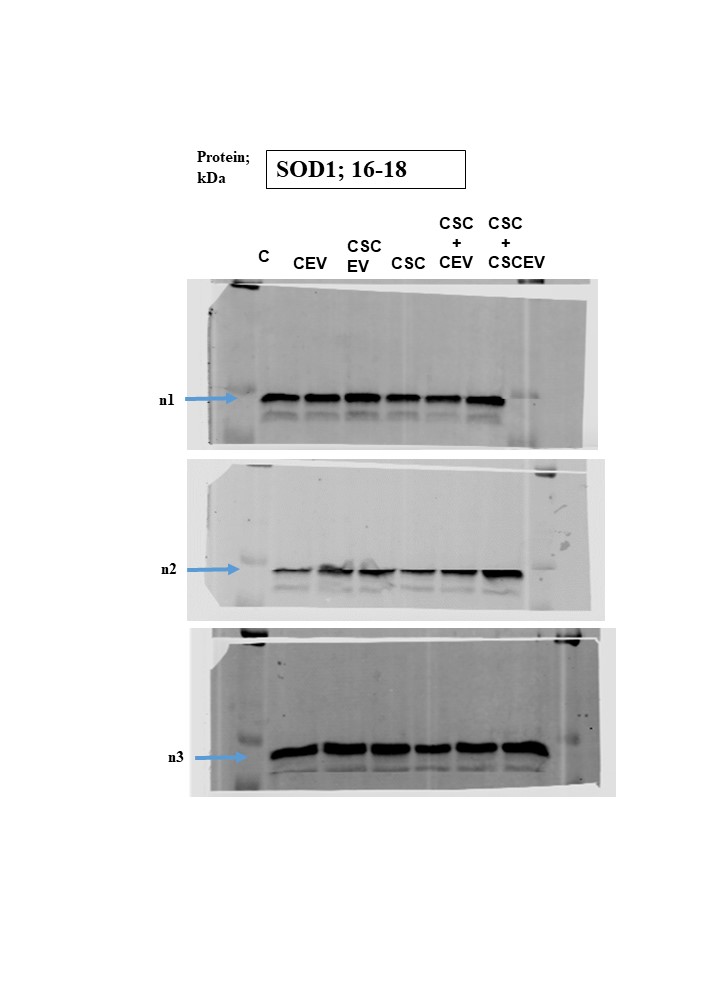


**Supplementary Figure S5:** **Figure 6 original blots**: Effect of EVs derived from uninfected macrophages on the levels of **actin** in SHSY-Y5 neuronal cells. ‘n’ represents the number of replicates. CEV represents EVs isolated from the control group; CSCEV represents EVs isolated from the CSC-treatment group


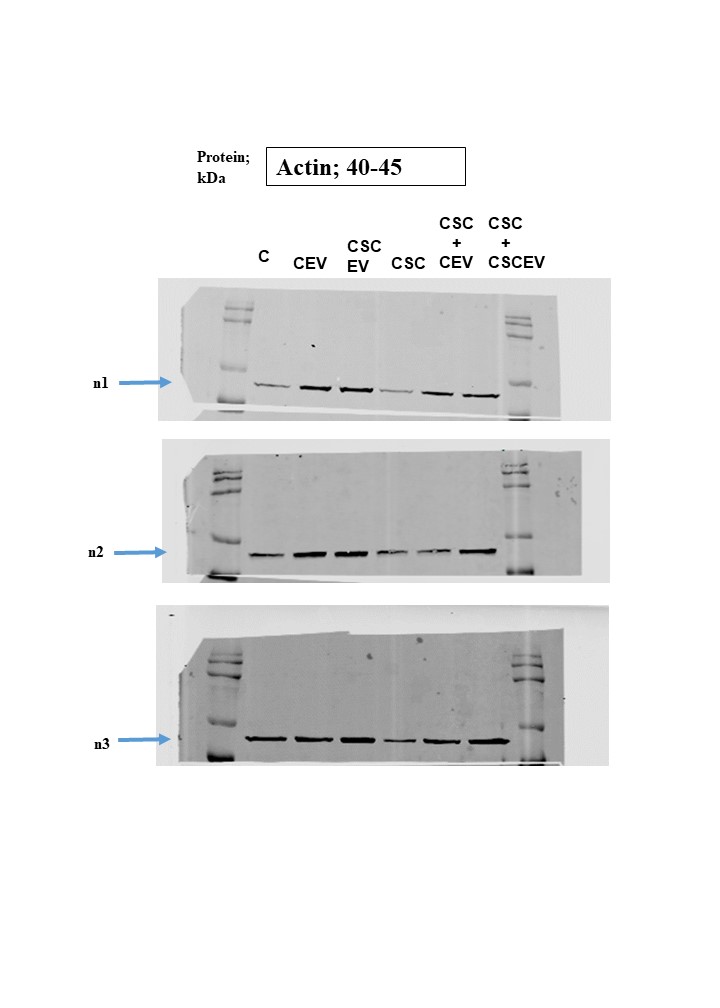


**Supplementary Figure S6:** **Figure 7 original blots**: Effect of EVs derived from uninfected macrophages on the levels of IL-1β in SHSY-Y5 neuronal cells. ‘ n’ represents the number of replicates. CEV represents EVs isolated from the control group; CSCEV represents EVs isolated from the CSC-treatment


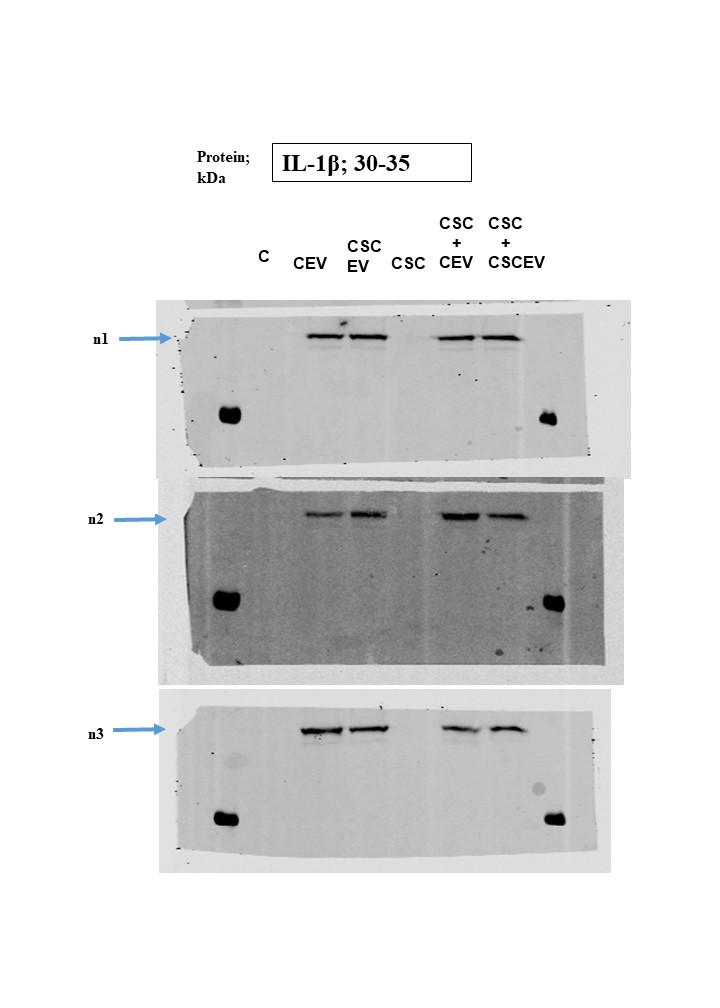


**Supplementary Figure S6:** **Figure 7 original blots**: Effect of EVs derived from uninfected macrophages on the levels of CYP2A6 in SHSY-Y5 neuronal cells. ‘ n’ represents the number of replicates. CEV represents EVs isolated from the control group; CSCEV represents EVs isolated from the CSC-treatment group


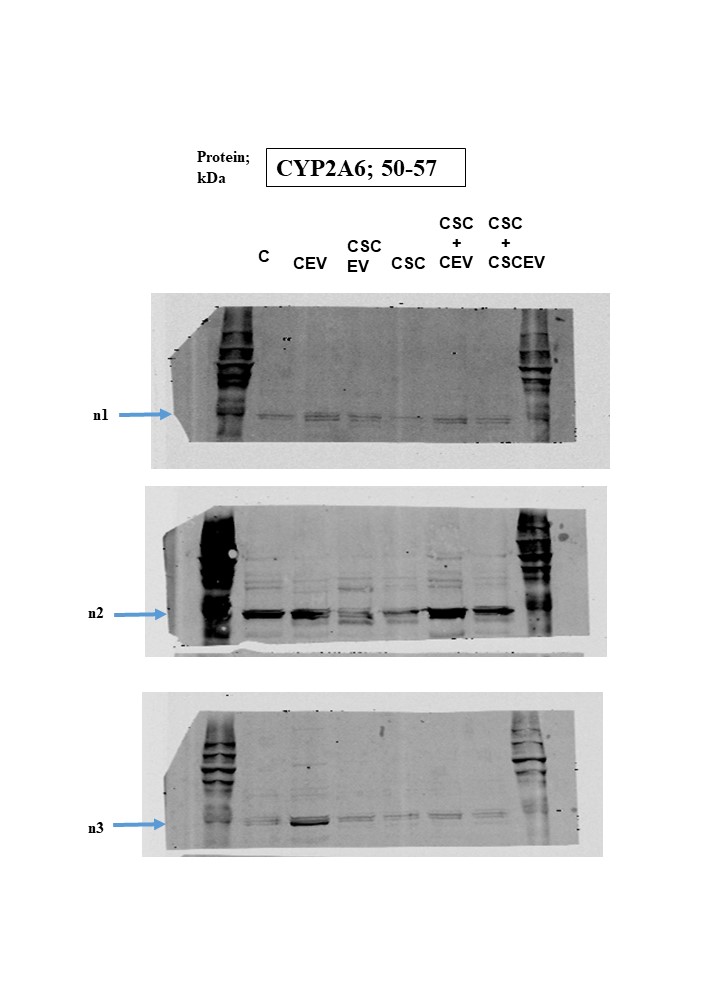


**Supplementary Figure S6:** **Figure 7 original blots**: Effect of EVs derived from uninfected macrophages on the levels of **Catalase** in SHSY-Y5 neuronal cells. ‘ n’ represents the number of replicates. CEV represents EVs isolated from the control group; CSCEV represents EVs isolated from the CSC-treatment group


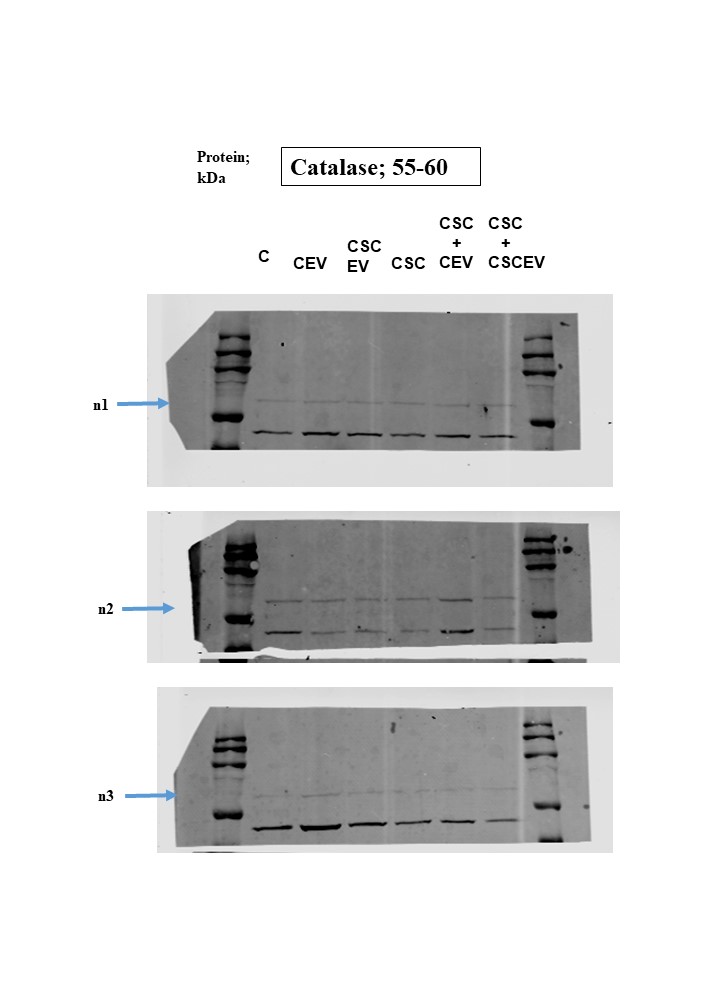


**Supplementary Figure S6:** **Figure 7 original blots** Effect of EVs derived from uninfected macrophages on the levels of **SOD1** in SHSY-Y5 neuronal cells. ‘ n’ represents the number of replicates. CEV represents EVs isolated from the control group; CSCEV represents EVs isolated from the CSC-treatment group


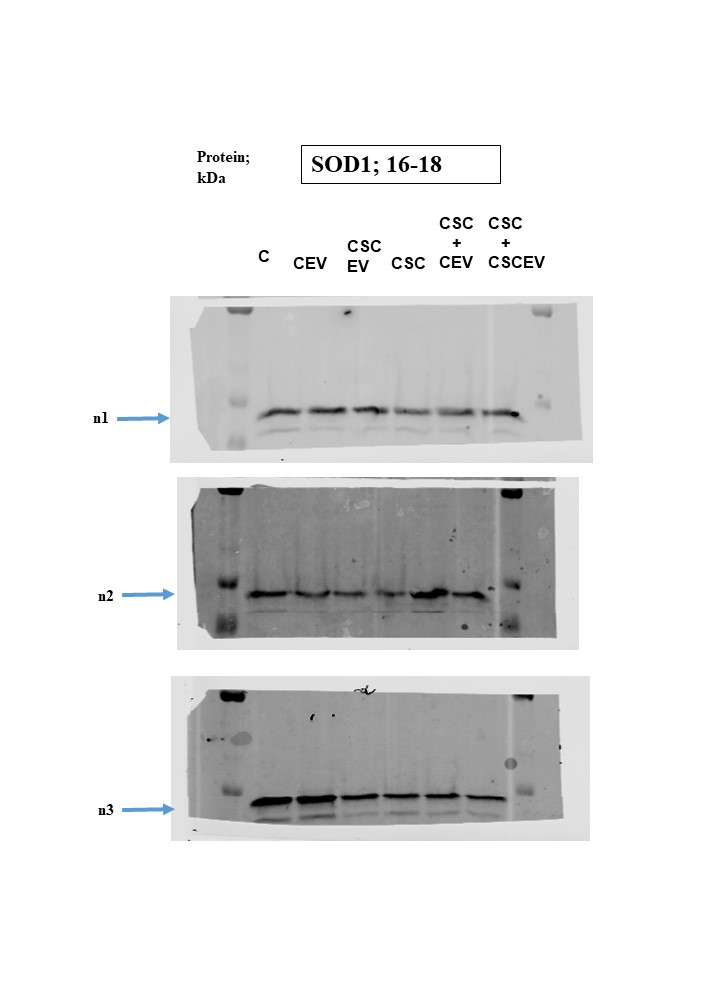


**Supplementary Figure S6 :** Figure 7 original blots : Effect of EVs derived from uninfected macrophages on the levels of **actin** in SHSY-Y5 neuronal cells. ‘ n’ represents the number of replicates. CEV represents EVs isolated from the control group; CSCEV represents EVs isolated from the CSC-treatment group


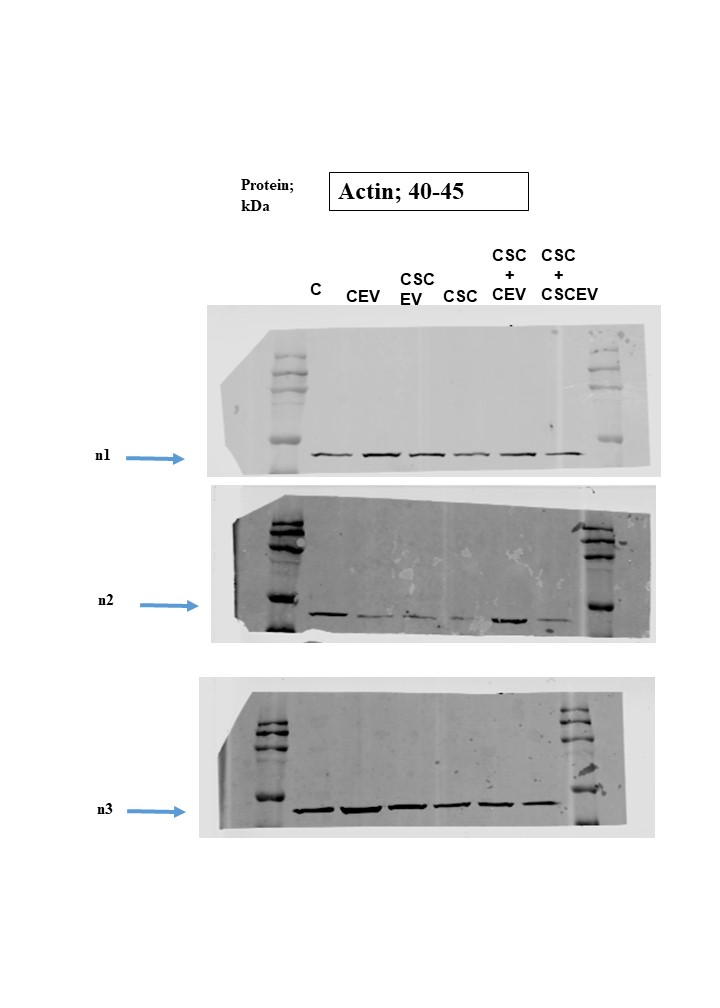


**n3**
